# Shape Memory Elastomers: A Review of Molecular Structures, Stimulus Mechanisms, and Emerging Applications

**DOI:** 10.1021/polymscitech.4c00035

**Published:** 2025-03-25

**Authors:** Yuan Yuan Yu, Hong Ping Xiang, Long Fei Fan, Ming Qiu Zhang

**Affiliations:** † Guangdong Provincial Key Laboratory of Functional Soft Condensed Matter, School of Materials and Energy, Guangdong University of Technology, 510006 Guangzhou, Guangdong, China; ‡ College of Textile Science and Engineering, 47892 Wuyi University, 529020 Jiangmen, Guangdong, China; § Key Laboratory for Polymeric Composite and Functional Materials of Ministry of Education, GD HPPC Lab, School of Chemistry, 26469 Sun Yat-Sen University, 510275 Guangzhou, China

**Keywords:** shape memory effect, shape memory elastomer, stimulus-response mechanism, smart polymer

## Abstract

Shape memory elastomers, a class of smart polymers, can remember their initial shapes under external stimuli and have potential applications in many fields, including medical devices, artificial muscles, actuators, and soft robots. Therefore, this review aims to explore research progress on the molecular structures, stimuli-responsive mechanisms, and emerging applications of shape memory elastomers and their composites in recent years. First, the molecular structures, shape memory effects, and working mechanisms of shape memory elastomers are thoroughly discussed and explained based on different external stimuli, including heat, light, electricity, magnetic fields, and solvents. Subsequently, emerging applications of shape memory elastomers, such as artificial muscles, actuators, soft robots, smart electronics, and aerospace, are presented. Finally, future challenges and insights into shape memory elastomers are discussed. Shape memory elastomers have received extensive attention and show great application potential in many emerging fields.

## Graphical abstract

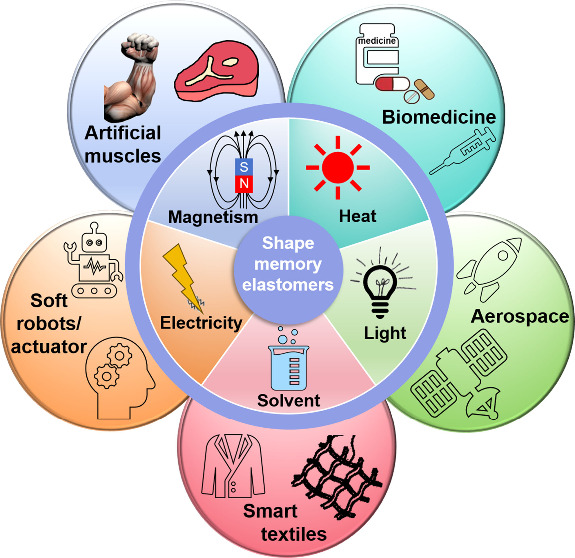

## 1 Introduction

Shape memory effect (SME) is a special behavior of materials that can “remember” a particular shape and return to the original shape from a programmed temporary shape when external stimuli such as heat, light, magnetism, electricity, or solvent are applied.^1–3^ Since their discovery of metal alloys in 1932, SMEs have been extensively investigated for their potential use in medicine, aerospace, and other fields.^4–9^ With the deepening development of material science and processing technology, SMEs have evolved from simple one-way SMEs to complicated two-way SMEs, and from single SMEs to triple SMEs and beyond.^10–14^ SMEs have been widely integrated into various alloys, ceramics, polymers, and composites for various applications under different stimuli.^15,16^

Elastomers, as defined by the American Society for Testing and Materials, refer to any natural or synthetic rubber-like polymers with both elastic and viscous characteristics that can quickly return to their original shape and size from substantial deformations after releasing external stress or force at room temperature. Their unique elasticity, flexibility, and insolubility provide exceptional properties, including excellent adhesion; abrasion resistance; resistance to chemicals, gas, water, and vapor; low glass transition temperature; and superior insulation capabilities.^17–20^ Thus, elastomers play an essential and ubiquitous role in industry and daily life and have been widely used in many large-scale applications, such as tires, construction, seals, adhesives, coatings, and damping absorbers.^21,22^

Intriguingly, the integration of SMEs into elastomers yields shape memory elastomers, which simultaneously have shape memory functions and unique viscoelastic properties. Compared to shape memory alloys, ceramics, and other polymers, shape memory elastomers generally have much better elasticity, flexibility, recovery rates, reliability, and shape memory cycles, allowing them to adapt to complex shapes, withstand large deformations, and maintain good mechanical properties and SME over multiple cycles.^23–25^ They show broad application prospects, including aerospace,^26^ biomedical devices,^27^ flexible electronics,^28^ intelligent textiles,^29^ soft actuators,^30^ soft robotics,^31^ and 4D printing.^32^ Therefore, a comprehensive review of the latest advances in shape memory elastomers will promote their rapid development and wide application in the future.

To date, some reviews of shape memory elastomers have been reported; however, they have focused on different aspects. Gopinath et al.^33^ summarized thermoresponsive shape memory elastomers according to their basis, design, and classification, as well as the characteristics of elastomers and polymer blends. Prathumrat et al.^34^ reviewed the synthesis, design, advanced manufacturing, and emerging applications of shape memory elastomers, mainly discussing the synthesis methods of dynamic covalent bond reactions, and introduced the application of liquid crystal elastomers to shape memory. Suethao et al.^35^ reviewed shape memory elastomers for biological applications, mainly covering self-healing shape memory elastomers, thermoplastic shape memory elastomers, and antibacterial and antifouling shape memory elastomers. Katzenberg et al.^36^ summarized shape memory elastomers prepared from natural rubber, including the ability to store extreme strain and cold energy, as well as the ability to perceive and remember environmental parameters. Reghunadhan et al.^37^ reviewed shape memory elastomers from the perspective of natural and synthetic rubber and discussed various application fields and scope for their future impact. Previous reviews of shape memory elastomers have covered specific aspects of materials and their applications. However, the comprehensive stimulus-response mechanisms of shape memory elastomers have not yet been fully explored. Different stimulus-response characteristics have different advantages and disadvantages, which determine their potential applications. Liquid crystalline elastomers exhibit outstanding SMEs; however, their complicated synthesis and high cost limit their widespread application. Therefore, this review aims to summarize and discuss recent advances in shape memory elastomers that utilize only traditional elastic materials as matrices according to their different stimulus-response mechanisms (Figure 1). This review systematically summarizes SMEs and working mechanisms of shape memory elastomers under different external stimuli and presents their emergent applications. Finally, the challenges and prospects of shape memory elastomers are discussed.

**Figure 1:**
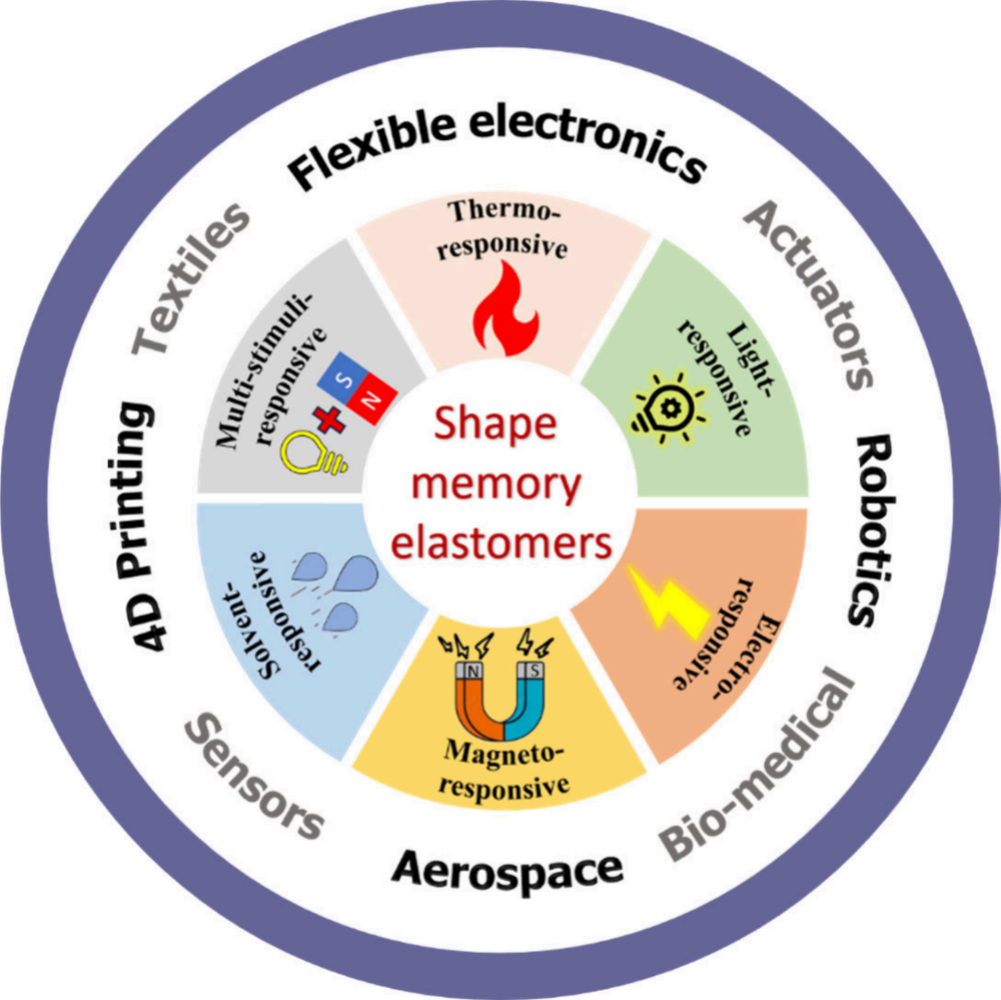
Overview of shape memory elastomers for this review.

## 2 Design of Shape Memory Elastomers

### 2.1 Shape Memory Mechanisms within Elastomers

The shape memory phenomenon depends directly on various factors such as the molecular structure, orientation, crystallinity, cross-linking density, intermolecular interactions, and programming process of polymers.^37–40^ The programming process is that the sample is mechanically deformed at a high temperature, typically 20 °C above the glass transition temperature (*T*_g_) or at the crystal melting temperature (*T*_*m*_), and then cooled below the switching temperature (*T*_*s*_ =*T_g_/ T_m_* ), where the molecular chain motion is frozen, and the polymers are in a higher energy state.^41–45^ Once frozen molecular chains are reactivated by external stimuli such as heat, light, or electricity, entropic energy is released, and molecular chains are induced to revert to their lowest energy state, causing restoration of the original “memory” shape from the temporary shape.

Elastomers often have partially cross-linked networks, whereas other thermosetting polymers feature highly cross-linked networks, where the partially cross-linked structures allow them to behave similarly to amorphous polymers. For shape memory elastomers, two structural elements primarily determine their SME: cross-linking networks/netpoints (fixing phase) and reversible molecular segments (reversible phase), which are used to remember permanent and temporary shapes, respectively.^46–49^ The cross-linked network prevents the molecular chains from sliding, flowing, and creeping when deformed, thus remembering permanent shapes.^50–52^ The fixing phase comprises polymers that are physically or chemically cross-linked and characterized by a high *T*_*m*_ or *T*_g_.^53–56^ Chemical or physical cross-linking, crystallization, entanglement, and interpenetrating networks can act as netpoints.^57–62^ After programming, molecular chains with a low *T*_g_ or crystalline regions with a low *T*_*m*_ typically serve as reversible phases. This allows changes in the molecular structure, inducing crystallization/melting phase transition or glass/rubber phase transition.^63–66^ Elasticity shifts when polymers are heated above their switching temperature, and conformational changes occur in amorphous regions (glass/rubber phase transition) or crystalline regions (crystallization/melting phase transition).^67–71^ Therefore, *T*_g_ or *T*_*m*_ is often used as the *T*_*s*_.^72–76^

In summary, the molecular structure of shape memory elastomers is composed of netpoints (fixing phase, high *T*_g_ /*T*_*m*_) and switching units (reversible phase, low *T*_g_/*T*_*m*_). Therefore, the fixing phase and the reversible phase are constructed by means of a multicomponent system design. Notably, different components must have good compatibility and different transition temperatures (*T*_g_/*T*_*m*_). The molecular chain responsible for the actuation remains in a partially oriented conformation, so the actuation behavior of shape memory elastomers is constrained by the geometric shape of the crystalline structure.^15^ Therefore, the shape memory elastomers can have excellent actuation performance by adjusting the orientation of the molecular chain, the coordination of the amorphous region and the crystalline region, and introducing reversible bonds.^15,172^ In addition, cross-linking density should also be considered, because cross-linking will reduce conformational entropy and increase the constraint on the movement of the molecular chain, thus affecting *T*_g_.^173–175^ Chemical and physical cross-linking can be used to generate shape memory elastomers to provide stability and determine permanent shape.^176,177^ Therefore, cross-linking should be fully considered in molecular structure design. Wang et al.^178^ systematically studied the effects of molecular weight of soft segment on hydrogen bonding, thermal properties, microphase separation, and mechanical properties of shape memory elastomers. The results showed that with the increase of the molecular weight of the soft segment, the degree of hydrogen bonding decreased, microphase separation weakened, and the *T*_g_ of the soft segment and the *T*_*m*_ of the hard segment roughly decreased. Moreover, the molecular weight and distribution should also be considered. The distribution of molecular weight affects fluidity, processability, and final shape memory properties.^179,180^ The molecular weight distribution must be reasonably controlled. Through the above comprehensive considerations, the deformation, retention, and recovery of the molecular structure under the applied force and external activation can be realized.

The working mechanism of SME in thermoresponsive shape memory elastomers is triggered by direct heating. Light-responsive shape memory elastomers achieve the SME by introducing light-sensitive groups or light-thermal fillers into the elastomer matrix to induce a thermally triggered SME.^77^ When electricity passes through conductive elastomers, the generated Joule heat activates the SME. Another way to trigger the SME of elastomers is by applying alternating magnetic fields, where ferromagnetic fillers are composited with elastomers to form magneto-responsive elastomers.^78^ The SME of solvent-responsive elastomers stems mainly from the molecular-structure design of dynamic networks or hydrogen bonding,^79–81^ and some of them require heat to assist in completion. Table 1 shows that many shape memory elastomers with different stimulus-response mechanisms have been developed, including heat, light, electricity, magnetism, solvent.

**Table 1:** Stimuli-Response Types, Shape Memory Effect, and Properties of Shape Memory Elastomers

		Shape memory effect		Performance	
Stimuli types	Elastic matrices	One-way	Two-way	*R*_*r*_ (%)	*R*_*f*_ (%)
Thermoresponsive	1. Thermoplastic polyurethane, epoxidized natural rubber and polymerized zinc dimethacrylate^82^	●^a^	○^b^	98	95
	2. Ethylene octene copolymer and ethylene propylene diene terpolymer^39^	●	○	92.1	86.7
	3. Hindered phenol/acrylic rubber^40^	●	○	99	99
	4. Poly(methyl methacrylate) and natural rubber^48^	●	○	98	98
	5. Amino-terminated oligomeric polyamide-1212 and isocyanate-terminated polyurethane^130^	○	●	96.6	86.7
	6. Eucommia ulmoides gum and Benzene-1,4-diboronic acid^131^	○	●	98	97
Light-responsive	**Graphene as filler:**				
	1. Polyurethane/functionalized reduced graphene oxide composites^85^	●	○	92.3	94.1
	2. Diselenide-containing polyurethane and graphene oxide^86^	●	○	95.5	96.7
	**MXene as filler:**				
	3. Hexamethylene-diisocyanate trimer and a functionalized MXene^87^	●	○	97.8	88.1
	**Nanotubes as filler:**				
	4. Poly(vinyl alcohol) and carbon nanotubes^88^	●	○	96.4	90.2
	5. Shape memory polyurethane and lignin nanotubes^89^	●	○	98.1	98.8
Electro-responsive	**Carbon nanotubes as filler:**				
	1. Thermoplastic polyurethane, polylactide and multi-walled carbon nanotubes^90^	●	○	85	95
	2. Carboxylated nitrile rubber and multi-walled carbon nanotubes^91^	●	○	>90	>90
Magneto-responsive	**Fe**_**3**_ **O**_**4**_ **as magnetic nanoparticle (MNP):**				
	1. Polyurethane and modifying Fe_3_O_4_ with octadecyl isocyanate^94^	●	○	>95	>95
	2. Fe_3_O_4_, cellulose nanofibers and poly-hydroxybutyrate/poly(ε-caprolactone)^95^	●	○	>95	100
	3. Fe_3_O_4_, polydopamine nanoparticles, thermoplastic polyurethane^96^	●	○	82.3	94.1
	**Carbonyl iron particles as MNP:**				
	4. Thermoplastic polyurethane, polycaprolactone and carbonyl iron particles^97^	●	○	>95	>95
Solvent-responsive	**Solvent-responsive shape memory composites:**				
	1. Polyvinyl alcohol and graphitic carbon nitride^99^	●	○	>80	>90
	2. Telechelic-hydroxylated polyhydroxyalkanoate and polyethylene glycol (PEG)^98^	●	○	>90	>99

a ●: Applicable.

b ○: Not applicable.

### 2.2 Shape Memory Effect and Performance

Based on the number of temporary and original shapes, shape memory elastomers can be classified as dual shape memory effect (D-SME), triple SME (T-SME) or multiple SME (M-SME).^101^ Moreover, shape memory elastomers can be divided into one-way SME (1W-SME) and two-way SME (2W-SME), depending on whether the shape memory process is reversible. Traditional one-way shape memory elastomers with a single reversible phase can achieve only a temporary shape. Upon heating, they revert to their original shape, which is a well-known D-SME.^102–104^ The introduction of two reversible phases into elastomers can achieve a T-SME,^105^ which exhibits two temporary shapes during the recovery process. Similarly, more than three reversible phases and three temporary shapes are considered M-SME. The realization of an M-SME is more difficult than that of a T-SME by increasing the number of reversible phases and temporary shapes, because more distinct crystallization/melting transitions or glass transitions are involved, and the content of each reversible phase is inevitably reduced as more types of reversible phases are introduced, leading to a decrease in the shape fixation rate of each temporary shape.^106–110^

The shape programming processes for 1W-SME and 2W-SME are similar. They are deformed above their respective transition temperatures and subsequently locked into a deformed shape during cooling (Figure 2). After programming, 1W-SME can restore their initial shape when activated by various stimuli, such as heat, light, electricity, and solvents, but they are unable to transform into a temporary shape from a permanent shape.^111,112^ To achieve a 2W-SME, the switching phase (crystalline region or liquid crystal) in the elastomer must cooperate with the internal/external stress to achieve reversible deformation during the temperature change. 2W-SME can switch reversibly between two shapes after programming.

**Figure 2:**
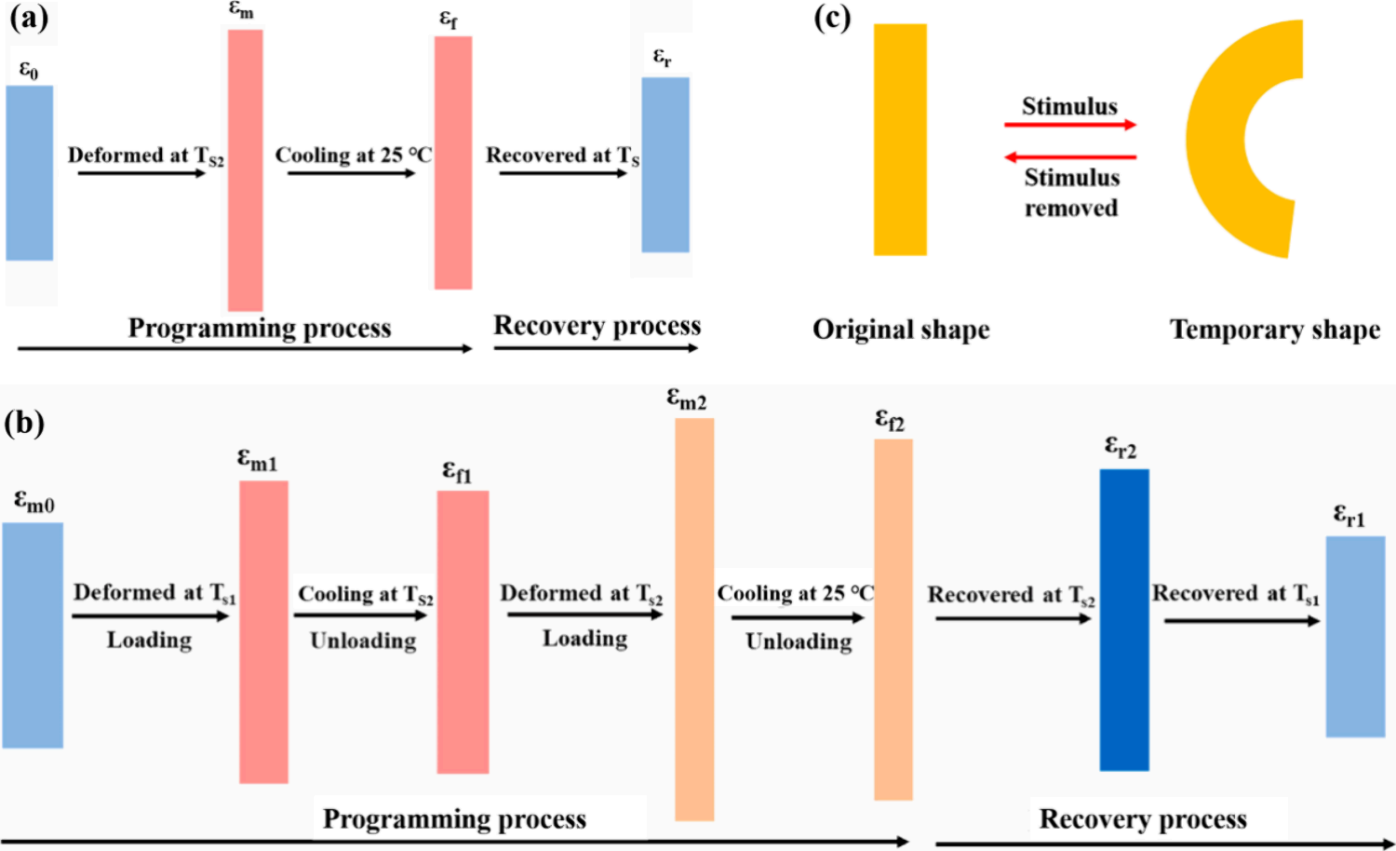
Schematic diagram of SME. 1W-SME: (a) D-SME. (b) T-SME. (c) 2W-SME. (a, b) Adapted with permission from ref 112. Copyright 2022 Elsevier.

For 1W-SME, the shape memory fixation rate (*R*_*f*_) refers to the ratio of elastomers that successfully achieves the target shape during shape memory fixation. *R*_*f*_ represents the ability of the elastomers to maintain the intended shape precisely during programming. The shape memory recovery rate (*R*_*r*_) represents the proportion of elastomers that effectively return from their fixed shape to their original shape upon activation. *R*_*r*_ reflects the ability of the elastomers to accurately restore their original shape. Thus, *R*_*f*_ and *R*_*r*_ are often used to estimate the shape memory performance of shape memory elastomers, and can be calculated using eqs 1 and 2, respectively.^9^

1 $$R_{f}=\frac{\varepsilon_{u}}{\varepsilon_{m}}\times 100\%$$

2 $$R_{r}=\frac{\varepsilon_{u}\left(N\right)-\varepsilon_{p}\left(N\right)}{\varepsilon_{u}\left(N\right)-\varepsilon_{p}\left(N-1\right)}\times 100\%$$

where ε_*u*_ represents the strain that remains fixed in the specimen after stress relaxation, ε_*m*_ is the maximum strain applied, ε_*p*_ is the residual strain following the recovery process, and *N* denotes the number of cycles.

*R*_*f*_ and *R*_*r*_ can also be obtained by measuring the angle:^113–115^

3 $$R_{f}=\frac{180^{{}^{\circ}}-\varphi }{180^{{}^{\circ}}}\times 100\%$$

4 $$R_{r}=\frac{\theta }{180^{{}^{\circ}}}\times 100\%$$

where φ is the recovery angle fixed at room temperature, and θ is the recovery angle under the external stimulus.

For 2W-SME, the average reversible strain during the heating and cooling cycles is typically used to characterize them.

## 3 Stimulus Response Types of Shape Memory Elastomers

### 3.1 Thermoresponsive Shape Memory Elastomers

Thermoresponsive shape memory elastomers have garnered significant attention as one of the most researched types of response and are often sensitive to thermal stimulation.^116,117^ Thermoresponsive shape memory elastomers can reversibly transform their shapes and sizes at different temperatures. Thermal stimuli are among the most commonly used, fundamental, and straightforward stimuli for shape memory elastomers. Thermoresponsive shape memory elastomers are also referred to as thermoinduced or thermoactuated shape memory elastomers. When thermal energy from the external environment acts on shape memory elastomers, it is transmitted in a direct contact manner through conduction, convection, radiation, etc.

Generally, the dual shape memory effect requires only one transition temperature (*T*_g_/*T*_*m*_) to achieve shape memory recovery. Triple shape memory effect has two temporary shapes, requiring two transition temperatures (*T*_g_/*T*_*m*_) to achieve shape transformation. As the number of shapes in memory increases, the number of transition temperatures required increases, and molecular design becomes more difficult. Therefore, there are few reports on multiple shape memory elastomers. In addition, ionomers with broad *T*_g_ can exhibit a tunable shape memory effect. Its specific feature is that these ionomers only have a wide range of reversible phase transitions, which can exhibit D-SME, T-SME and M-SME.^182^

If there is no additional description such as dual, triple or multiple, shape memory elastomers belong to 1W-SME.^118^ When the crystalline region in a shape memory elastomer used as the reversible phase melts, shape recovery of the shape memory elastomers is triggered.^119^ In a dual shape memory effect, when the temperature reaches *T*_*S*_, shape memory elastomers transform from their temporary to initial shape.

Two or more types of polymers can be blended to form new shape memory composites with better mechanical properties. The core principle involves one polymer in a fixed phase and the other in a reversible phase. Aiswarya et al.^82^ employed dynamic vulcanization to blend polymerized zinc dimethacrylate, thermoplastic polyurethane (TPU), and epoxidized natural rubber (ENR) to create a novel shape memory elastomer (Figure 3a). TPU/ENR could be transformed between its permanent and temporary shape at a transition temperature (>80 °C) (Figure 3b). Shape recovery of TPU/ENR blends was similar to that of pure TPU, demonstrating excellent shape memory behavior (*R*_*f*_ was approximately 95%, and *R*_*r*_ was approximately 98%) at the optimal *T*_*S*_ (Figure 3c, d).

**Figure 3:**
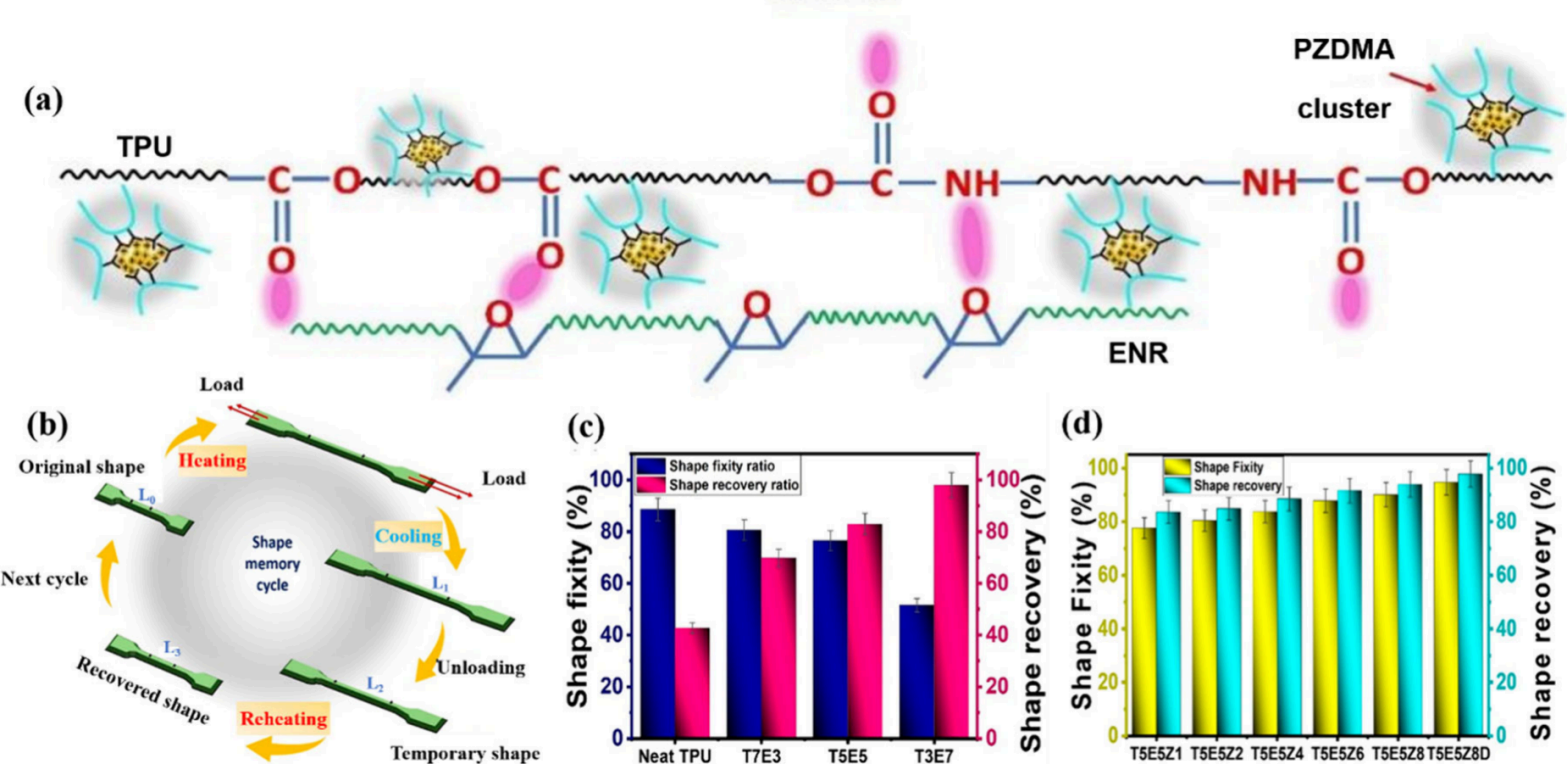
(a) Zinc dimethacrylate and the TPU/ENR blend. (b) TPU/ENR blend of SME. (c) SME of pure TPU and TPU/ENR blends at various ratios. (d) Shape fixity and recovery performance of TPU50/ENR50 with varying ZDMA content. (a–d) Reproduced from ref 82. Copyright 2023 American Chemical Society.

There are other methods for preparing D-SME shape memory elastomers. Fu et al.^120^ reported the synthesis of poly­(ether-*b*-amide) (PEBA) via a two-step melt polycondensation using PA1212 and polytetramethylene ether glycol (PTMEG). The SME of PEBA could be actuated by the crystallization/melting of PTMEG and the microphase separation between the PA1212 and PTMEG domains. PEBA-12 exhibited an obvious SME (*R*_*f*_: 92%, *R*_*r*_: 98%), and *R*_*f*_ and *R*_*r*_ of PEBA-22 remained 100% and 90%, respectively, after five cycles. Sun et al.^121^ developed heat-triggered shape memory elastomers with a sea–island structure, consisting of an ethylene-acrylic acid copolymer (EAA) and nitrile-butadiene rubber (NBR), through peroxide-induced dynamic vulcanization. The EAA/NBR has outstanding *R*_*f*_ (>91%) and *R*_*r*_ (>93%) values at a temperature of 95 °C. Zhang et al.^122^ introduced tea polyphenols (TP) onto carbon black (i.e., TP@C) using the vacuum absorption method and subsequently added metal ions to the rubber matrix. With the introduction of TP@C and ZnCl_2_ to construct a dynamic coordination bonding structure, the rubber composite exhibited excellent solid plasticity. The NBR/TP@C composite exhibited excellent and stable shape memory properties (*R*_*f*_: 93.5%, *R*_*r*_: 92.4%). Lin et al.^123^ prepared shape memory elastomers from epoxidized natural rubber and zinc diacrylate (ZDA) via the Oxa-Michael reaction. The SME was achieved using *T*_g_ (*T*_g_ changed with the addition of ZDA, ranging from 20 to 46 °C) as the transition temperature. When it was reheated above *T*_g_, the elastic entropy was released and the shape is restored to its original shape (*R*_*f*_: ∼100%, *R*_*r*_: 95-100%; shape recovery time was between 12 and 33 s). In addition, they also prepared a reversible plastic shape memory elastomer by incorporating the aggregates of deformable glassy hindered phenol (AO-80) into the epoxidized natural rubber (ENR) and ZDA systems within the amorphous network to adjust the *T*_g_ of the system.^124^ The sample was deformed, fixed at 25 °C and then heated to 80 °C to restore the original shape (*R*_*f*_: 75.6%, *R*_*r*_: 96.0%). Research showed that incorporating compatible low molecular weight additives into the amorphous network was expected to become a common method for manufacturing reversible plastic shape memory elastomers.

The design and preparation of T-SME and M-SME, which differ only in the number of shapes in the recovery process, are more challenging than those of D-SME. Xin et al.^125^ investigated the T-SME of cross-linked trans-polyisoprene/poly­(ethylene-*co*-vinyl acetate) (TPI/EVA) composites by varying the amount of dicumyl peroxide. The two phases were connected by chemical bonds, enabling the regulation of different shapes through distinct crystallization regions. At room temperature, the TPI/EVA composites exhibited three distinctive regions: crystallization, cross-linking, and amorphous domains (Figure 4a). Shape fixation was determined by the crystalline region, whereas the speed of shape recovery was influenced by the cross-linking density. As demonstrated in Figure 4b, these samples deformed to a “V” and “7” shape at 55 °C and were fixed to a “spiral” and “2” shape at 0 °C. Subsequently, they reverted to their initial shape upon heating. This research demonstrated that TPI/EVA composites exhibited an outstanding T-SME. The shape memory performance of sample T/E-D1.6, including its shape recovery rate and shape fixation rate, approached 100%.

**Figure 4:**
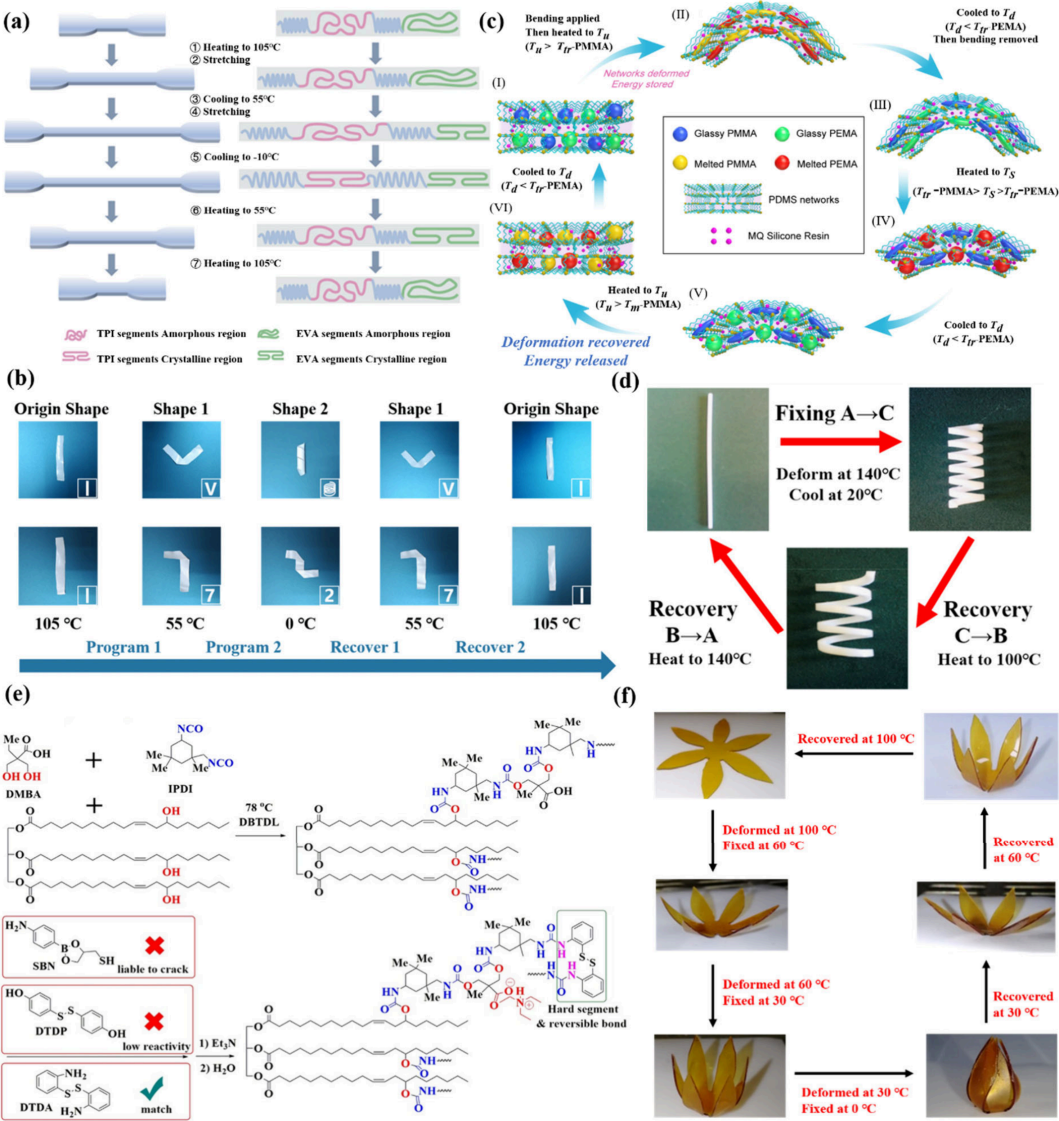
(a) TPI/EVA composites with T-SME. (b) T-SME of T/E-D0.4 composites. (c) Mechanism for designing the second temporary shape of SRCs. (d) T-SME of SRCs. (e) Synthesis of DTDA/castor-oil-based WPU dispersions. (f) Quadruple SME of PU-DTDA20. (a, b) Reproduced from ref 125. Available under a CC-BY 4.0 license. Copyright 2022 Xin et al. (c, d) Adapted with permission from ref 126. Copyright 2020 Elsevier. (e, f) Adapted with permission from ref 128. Copyright 2020 Wiley.

Huang et al.^126^ reported a straightforward approach involving blending monomers/prepolymers and in-situ polymerization/cross-linking to create shape memory silicone rubber composites (SRCs) featuring three distinct *T*_*S*_’s (*T*_*c‑PDMS*_ = −42 °C, *T*_*g‑PEMA*_ = 90 °C and *T*_*g‑PMMA*_ = 130 °C, as shown in Figure 4c). Figure 4d shows the T-SME of the prepared SRCs, and the shape fixation rate and shape recovery rate were 94 ± 0.5% and 95 ± 7.6%, respectively. Maimaitiming et al.^127^ prepared triple shape memory elastomers via radiation-induced vulcanization. Their superior T-SME stemmed from their heterogeneous macromolecular structure, oriented crystals, and distinct multiphase morphology. Thus, the entropy driven elasticity of discrete polypropylene (PP) and polyolefin elastomer (POE) switching segments achieved excellent *R*_*r*_ (88% and 94%). This was facilitated by reversible aggregation and solidification of PP and POE at *T*_*S*_ (90 and 180 °C).

Quadruple SMEs exhibit diverse shape changing capabilities. Research on quadruple SMEs is still in the exploratory stage, preparation methods are still evolving, and application fields are gradually expanding. Zhang et al.^128^ developed a high-strength multifunctional castor-oil-based waterborne polyurethane (WPU) by incorporating precise amounts of dithiodiphenylamine (Figure 4e). A wide glass transition temperature range enabled the films to exhibit versatile shape memory effects. The WPU film showed a maximum recovery (100%) of its original mechanical properties after undergoing up to four reprocessing cycles. PU-DTDA20 was selected to visually demonstrate quadruple SME (Figure 4f). After being reheated to a fixed temperature, the film retained its shape for over 30 min. In addition, Bila et al.^129^ reported two-component PU-urea networks that exhibit quadruple shape memory. They showed the feasibility of designing reprocessable poly­(urethane-urea) networks with multiple functionalities including quadruple SME. This was achieved by using a dynamic covalent bond (urethane bond exchange). This study provided new ideas for the preparation of quadruple-shaped memory elastomers.

As the variety of shape memory elastomers increases, it becomes possible to acquire two or even three temporary shapes in single shape memory elastomers. 2W-SME materials can be transformed into two different shapes, and shape transformation can be achieved by changing the temperature under external stress.^84^ In addition, 2W-SME achieves reversible and spontaneous shape changes between initial and temporary shapes after programming.

Li et al.^83^ synthesized a polyamide elastomer via polycondensation of a PA1212 prepolymer with polyetheramine. During cooling, when the temperature was within the melting range of the polyether soft domain, the oriented crystalline polyether soft domain underwent partial melting. The remaining unmelted soft domain, combined with the residual strain, generated internal stress that facilitates crystallization-induced elongation (Figure 5a). The soft polyether segments recrystallized under internal stress when the samples were cooled. During the heating and cooling cycles, the samples underwent spontaneous shape changes, demonstrating reversible T-SME. As shown in Figure 5b, at room temperature (∼25 °C), the strip sample first recovered from 0° to 145° and then automatically bent again to 110° at −20 °C. Then, when changing between room temperature and −20 °C, the alternating angles varied between 145° and 110° and exhibited a reversible response angle of about 35°, demonstrating excellent reversible 2W-SME performance.

**Figure 5:**
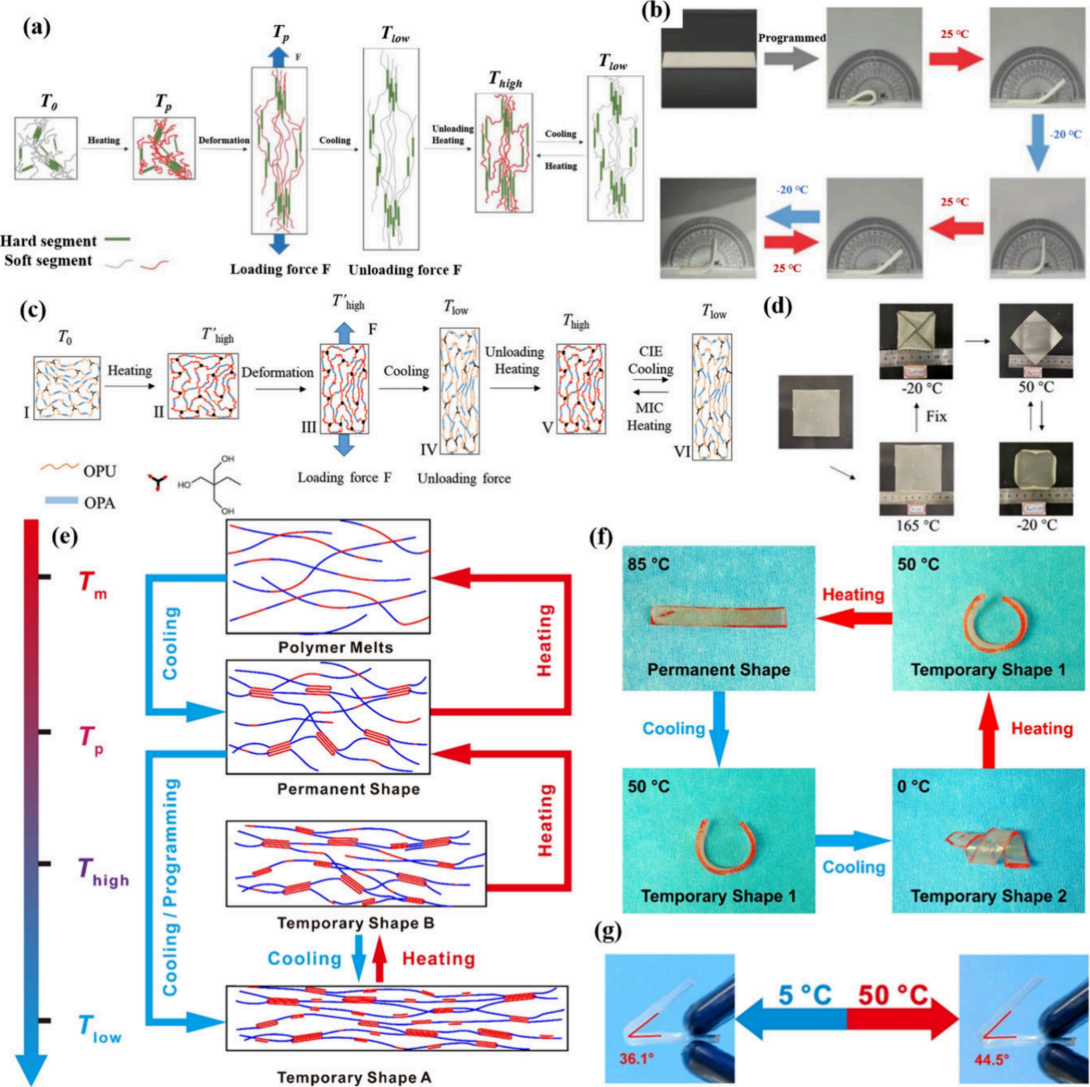
(a) Reversible SME of TPAE. (b) Reversible 2W-SME of T0.6-2. (c) Reversible 2W-SME for cPUUA–C7-S25. (d) Reversible 2W-SME of cPUUA–C7-S25. (e) Mechanism of the RSME. (f) Shape recovery process of E1 at 0, 50, and 85 °C. (g) Reversible SME. (a, b) Adapted with permission from ref 83. Copyright 2023 Wiley. (c, d) Adapted with permission from ref 130. Copyright 2022 Wiley. (e–g) Reproduced from ref 132. Copyright 2019 American Chemical Society.

Li et al.^130^ demonstrated the synthesis of poly­(urethane-urea-amide) elastomers (PUUAs), which contained a semi-crystalline block through the copolymerization of isocyanate-terminated polyurethane (OPU) with amino-terminated oligomeric polyamide-1212 (OPA). PUUAs had three types of transition temperatures (*T*_*c*_, *T*_*m*_ and *T*_g_) (Figure 5c), which enabled them to demonstrate versatile and reversible 2W-SME characteristics after programming (Figure 5d). Significant shape reversible transitions could be observed between −20 and 50 °C after shape programming at 165 °C and shape fixing at 20 °C. The *R*_*r*_ and *R*_*f*_ values were 96.6% and 86.7%, respectively.

Qi et al.^131^ reported the synthesis of multifunctional shape memory elastomers using *Eucommia ulmoides* gum to construct a dynamic cross-linking network. Because of their remarkable crystallization properties and broad melting-temperature range, these shape memory elastomers exhibited both 1W-SME and 2W-SME under constant stress or free of stress. A rectangular sample was folded at 60 °C and subsequently cooled to −20 °C to fix the temporary shape. Reheating to 38 °C enabled partial recovery of the initial shape. Reversible heating and cooling between 38 and -20 °C allowed reversible change in the bending angle between 31° and 46°. The broad Δ*T*_*m*_ indicated that the crystal domains have multiple sizes. Programming at a certain temperature (*T*_*part*_ = *T*_*m,onset*_ <*T*_*par*t_ <*T*_*m,end*_) leaded to melting of small crystal domains, resulting in partial restoration of the shape. The large crystal domains acted as structural frameworks, fixing shape A. Cooling to *T*_*c*_ allowed the growth of small crystal domains, fixing shape B owing to the crystallization-induced elongation effect. Reheating to *T*_*part*_ causes melting of the small crystal domains, resulting in the recovery of shape A, facilitated by the melting-induced contraction effect. Reversible heating and cooling between *T*_*part*_ and *T*_*c*_ resulted in reversible changes in shapes A and B. The sample exhibited properties with *R*_*f*_ and *R*_*r*_ valued over 97 and 98% after 4 cycles, reconfiguring at 140 °C for 30 min.

Notably, the first reversible shape memory elastomers made using available commercially thermoplastic polyolefin elastomers (TPE) were prepared by blending.^132^ Two types of reversible SME were prepared by blending POE Engage 8003 (POE1), Engage 8137 (POE2), Engage 8180 (POE3), and olefin block copolymer elastomer Infuse 9007, which had distinct melting temperatures. The mechanism of the reversible SME in the TPE blends was presented in Figure 5e. In Figure 5f, E1 (block copolymer elastomer (OBC):30 wt %, POE1:30 wt %, and POE3:40 wt %) was heated to 85 °C for 4 min, stretched at a prestress of 0.15 MPa, and subsequently cooled from 85 to 5 °C. After isothermal treatment at a fixed temperature of 5 °C for 3 min, the external stress was eliminated and fixed 76% temporary strain at 5 °C (*R*_*f*_ :95.6%). Upon heating back to 85 °C (*R*_*r*_:95.6%). Figure 5g shows the reversible shape memory effect between 5 and 50 °C.

### 3.2 Light-Responsive Shape Memory Elastomers

Thermoresponsive shape memory elastomers have garnered significant attention owing to their ease of handling, broad thermal-range compatibility, and environmental friendliness. However, thermoresponsive shape memory elastomers have drawbacks including challenges in precise control, achieving targeted deformation, and quantitative transformation. To address these issues, light-responsive shape memory elastomers have been developed. In addition, the working principle of photothermal shape memory elastomers is similar to that of thermoresponsive shape memory elastomers. The difference is that the light energy is converted into heat energy to actuate the SME. Light-responsive shape memory elastomers are often controlled by either photothermal effects or thermal photo-breakable network junctions.^133^ This necessitates integrating photo-absorbers such as carbon nanotubes into thermoresponsive shape memory elastomers.^134,135^ These shape memory elastomers are classified according to their differences in wavelength response.

Near-infrared light (NIR, 760 nm to 3 μm) is generally used as a shape memory response light source. When NIR light is applied to light-responsive shape memory elastomers, the SMEs are actuated by indirect heating by using photothermal conversion agents. Light-responsive shape memory elastomers are mostly added to photothermal conversion materials in the material matrix, which can trigger deformation when the prepared material reaches the transition temperature after a specific period of light irradiation. Li et al.^87^ constructed a PU containing a Diels–Alder (DA) interchangeable cross-linked structure utilizing a modified hexamethylene-diisocyanate trimer (B-THDI) and a functionalized MXene (FMXene), as shown in Figure 6a. FMXene exhibited a photothermal conversion effect. As shown in Figure 6b, the crystalline segments of the composites melted via the thermal conduction of FMXene, releasing stored strain energy and enabling recovery to their initial shapes under NIR light. A light-induced DA reversible cross-linking network was formed, resulting in an exceptional SME. This material exhibited a rapid shape recovery rate (<15 s) with shape fixity (*R*_*f*_ = 88.1%) and good recovery rates (*R*_*r*_ = 97.8%). This structure was capable of fully recovering its original configuration within 10 s of NIR-light irradiation. A 10 s irradiation of NIR light enabled PU/B-THDI/FMXene2 to reach its melting temperature (*T*_*m*_ = 70 °C) to achieve shape memory (Figure 6c).

**Figure 6:**
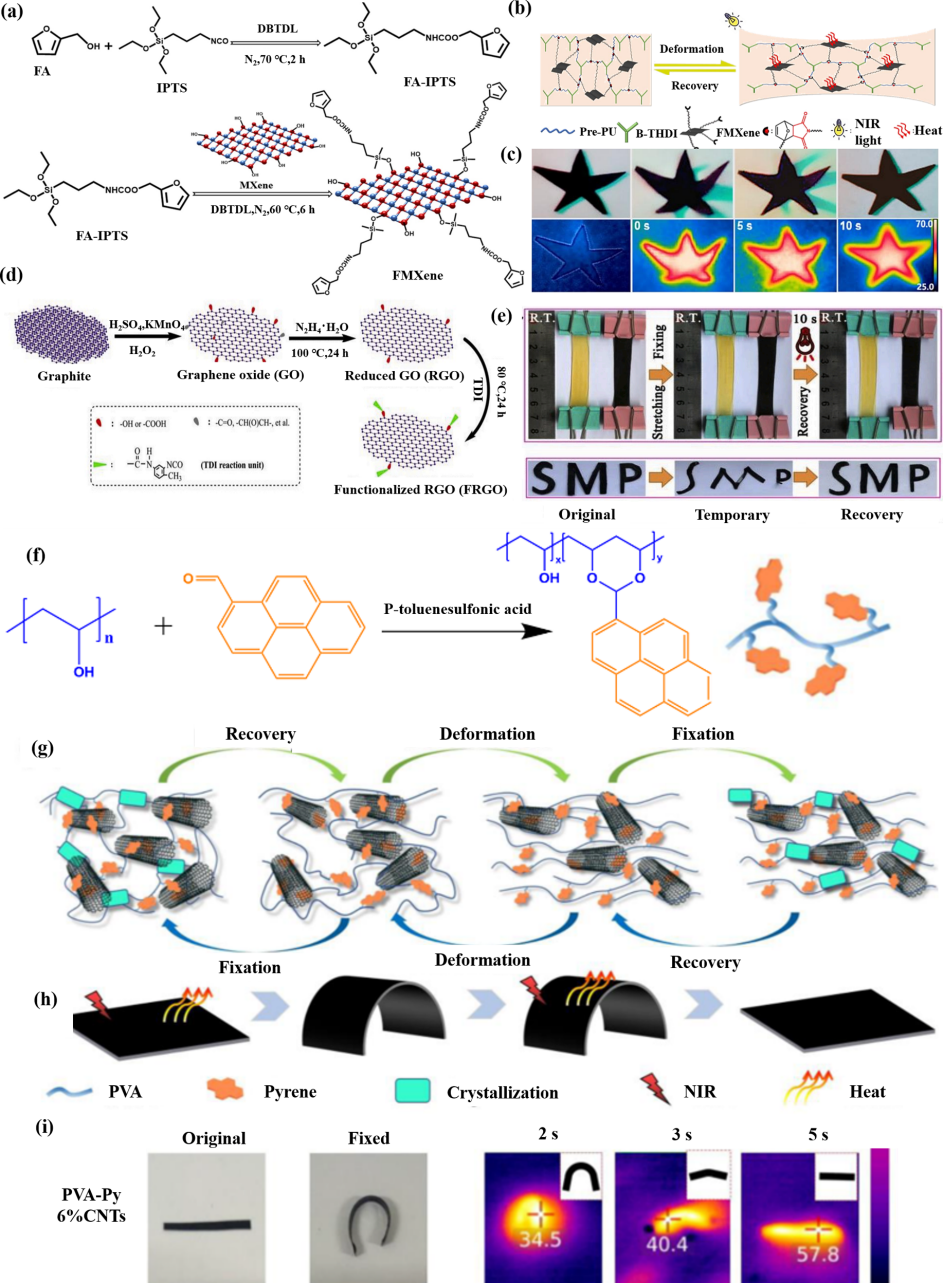
(a) Preparation process of FMXene. (b) Mechanism of shape memory by NIR light. (c) “Star-like” shape memory under NIR light. (d) Synthetic route for FRGO. (e) Shape memory processes of DAPU and their composite films. (f) Synthesis of PVA-Py; (g) PVA-Py/CNTs with a shape memory network. (h) Shape memory capability of PVA-Py/CNTs under infrared light. (i) Infrared thermal image of the light-responsive SME of PVA-Py 6%/CNTs. (a–c) Adapted with permission from ref 87. Copyright 2023 Elsevier. (d, e) Adapted with permission from ref 85. Copyright 2020 Elsevier. (f–i) Reproduced from ref 88. Copyright 2023 American Chemical Society.

Du et al.^85^ synthesized DA cross-linked polyurethane/functionalized reduced graphene oxide composites (DAPU-FRGOs). The synthesis route for the FRGO was shown in Figure 6d. Due to the photothermal effects of FRGO and the thermal reversibility of D–A bonds, the composites exhibited rapid shape recovery under NIR light (*R*_*f*_: 92.3% and *R*_*r*_: 94.1%) in less than 10 s. NIR light enabled DAPU-FRGO2 to reach its melting temperature (*T*_*m*_ = 50 °C) to actuate SME. First, the DAPU and DAPU-FRGO2 films in their original shapes were heated to approximately 50 °C. Then, they were stretched, which fixed their temporary shapes at -20 °C, whereas the deformed composite film was observed to recover its initial shape more rapidly upon exposure to NIR light (Figure 6e).

Dai et al.^88^ synthesized a conductive material with light-responsive shape memory properties. This material used the pyrene groups of poly­(vinyl alcohol) (PVA) as the polymer network and carbon nanotubes (CNTs) as nano-photothermal agents. A special type of polyvinyl acetate material with strong crystallization ability was used and further modified by introducing a phenyl group (Py), as shown in Figure 6f. Furthermore, the interaction between stacking and hydrogen bonds resulted in stable cross-linking, imparting an SME (Figure 6g). The CNTs exhibited a noticeable response to NIR irradiation (Figure 6h). Thus, the PVA-Py/CNT composites served as light-responsive shape memory elastomer systems, with the CNTs effectively functioning as light-absorbing fillers that convert light energy into heat. After exposure to NIR light, the prepared composite began to deform (Figure 6i). Shape recovery of the PVA-Py/CNT composites commenced within 1 s, reaching an *R*_*r*_ value of 81–98% after 5 s. The PVA-Py 6% CNTs exhibited excellent memory performance, with shape fixity of 96.4 ± 0.8% and shape recovery of 90.2 ± 0.9%.

Ultraviolet (UV) light with wavelengths ranging from 10 to 400 nm activates shape memory polymer composites containing photosensitive groups to exhibit a shape memory effect. UV radiation serves as an indirect heat source, facilitating the deformation of these shape memory polymer composites. The subsequent sections detail the operational principles of UV-driven shape memory polymer composites. Lignin nanotubes (LNTs) are commonly used as photothermal fillers. Wang et al.^89^ developed light-responsive composites using direct ink for 3D printing of shape memory polyurethane (PU) by incorporating UV-absorbent LNTs. PU/LNT composites had a photothermal conversion capability and could reach the glass transition temperature under UV irradiation to achieve the SME (Figure 7a). The shape recovery rate of the 3D-printed composites was as high as 99% under UV radiation (370 nm) for 15 min (Figure 7b).

**Figure 7:**
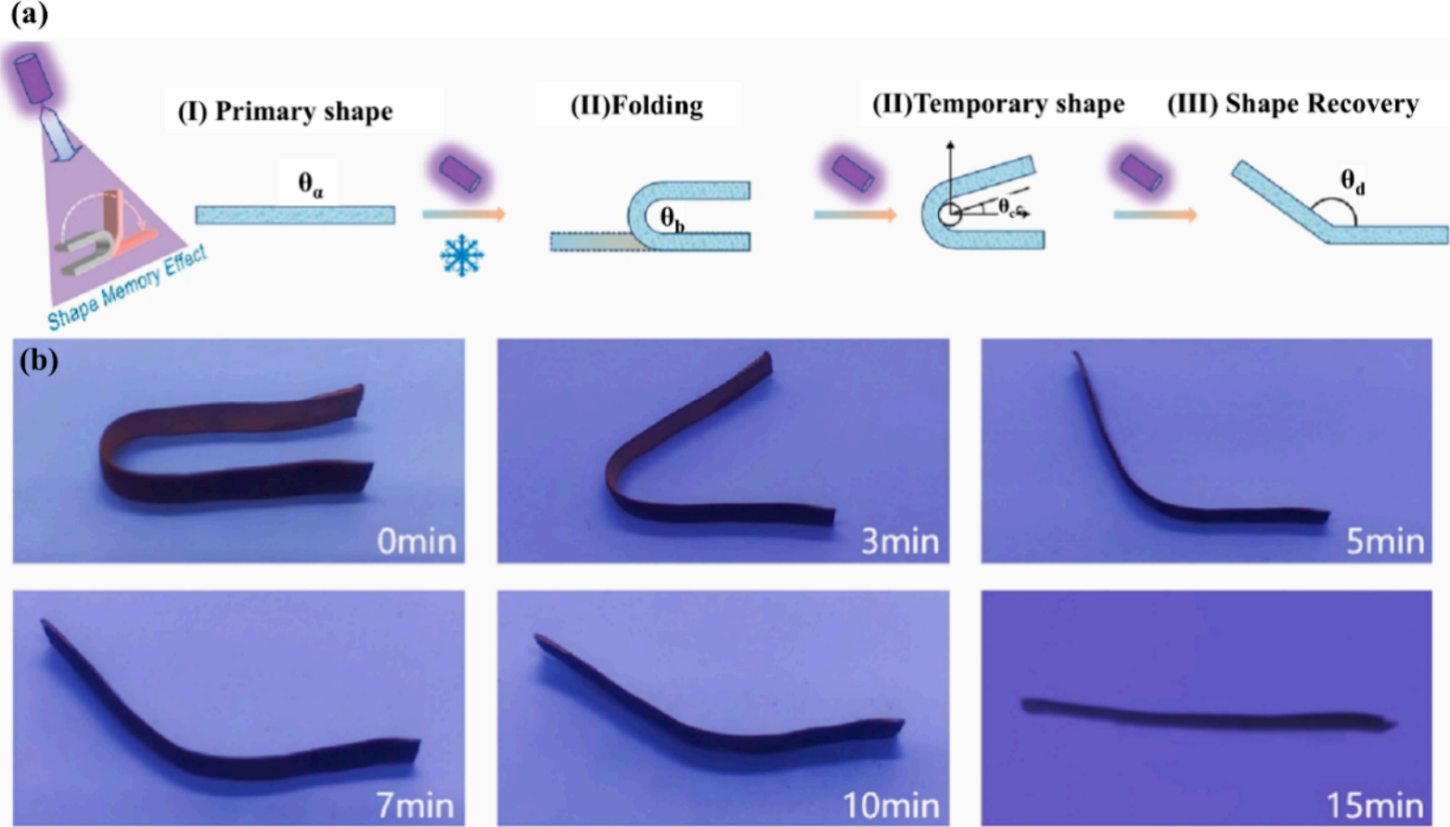
(a) Shape memory testing process. (b) 3D-printed composites of PU-5LNT. (a, b) Adapted with permission from ref 89. Copyright 2023 Elsevier.

Visible light (VIS) refers to the electromagnetic spectrum perceivable by the human eye, which typically lies in the wavelength range of 400 to 760 nm. Some studies have used the VIS-NIR wavelength range as the driving wavelength.

Du et al.^86^ fabricated multifunctional graphene oxide (mfGO) encapsulated with nitrogen-, phosphorous-, and silicon-containing units, and then integrated mfGo into diselenide-containing polyurethane (dPTD) to prepare composites (Figure 8a, b). The dPTD-mfGO2 composite was heated above its transition temperature (∼45 °C), subjected to shape programming, and then cooled to fix the temporary shapes. Upon exposure to VIS-NIR light, the spiral shape of the dPTD-mfGO2 composite returned to its initial shape after 10 s (Figure 8c). The *R*_*f*_ values of dPTB-mfGO2 and dPTD-mfGO2 films were 96.7% and 95.2%, respectively, and their *R*_*r*_ values were 95.5% and 93.1%, respectively. This was attributed to the photothermal effect induced by mfGO or GO, with NIR light playing a pivotal role in this enhancement (Figure 8d).

**Figure 8:**
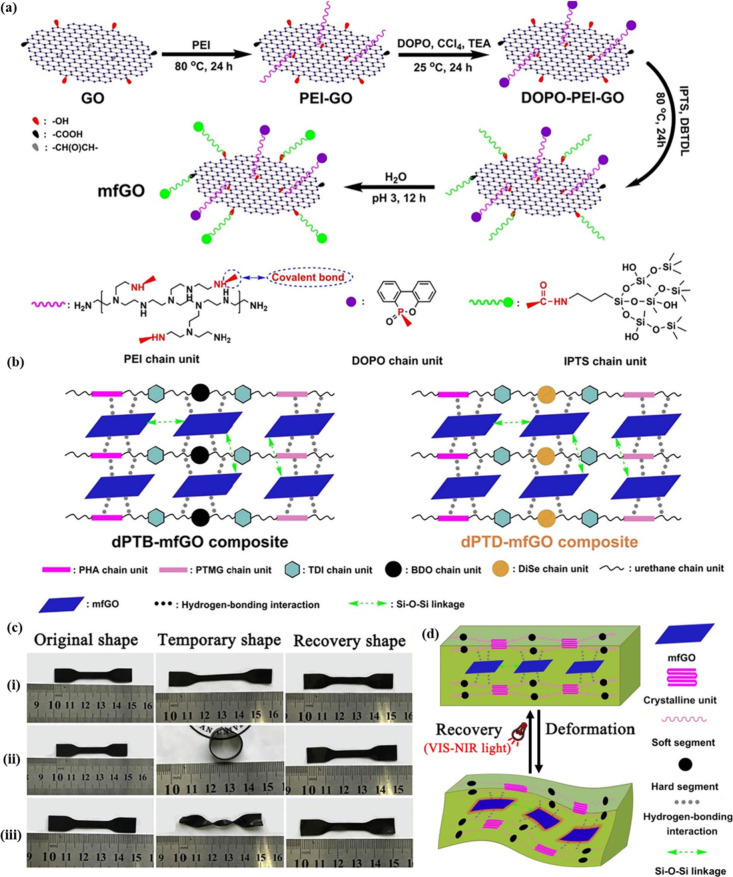
Synthesis routes of (a) mfGO. (b) dPTB-mfGO and dPTD-mfGO composites. (c) SME of dPTD-mfGO2. (d) Mechanism of VIS-NIR light-triggered SME. (a–d) Adapted with permission from ref 86. Copyright 2020 Elsevier.

### 3.3 Electro-Responsive Shape Memory Elastomers

Electro-responsive shape memory elastomeric composites are characterized by easy control, remote driving, and a fast response. Electro-responsive shape memory elastomeric composites are composite materials containing conductive fillers such as graphene oxide and carbon nanotubes. The conductive filler generates Joule heat through electrical energy and reaches the *T*_*S*_ to achieve SME. Liu et al.^90^ prepared electrically responsive shape memory elastomers by melt-blending thermoplastic polyurethane (TPU) and polylactide (PLA), and then multi-walled carbon nanotubes (MWCNTs) were added to the TPU/PLA blend system. When the MWCNTs were predominantly dispersed in the TPU phase, a double percolation network formed in the system, facilitating rapid electro-responsiveness. At a load of 20 V, TPU60/PLA40/MWCNT4 achieved a recovery rate of approximately 90%. The primary mechanism was that Joule heat generated by 20 V within 80 s caused the composite to reach a *T*_g_ that was higher than that of PLA (nearly 70 °C), which meet the requirements of TPU/PLA/MWCNT composites to achieve an electroactive SME.

González-Jiménez et al.^91^ produced shape memory elastomeric composites based on a carboxylic styrene–butadiene rubber matrix with both ionic and covalent cross-links supplemented with carbon black and MWCNTs as reinforcing fillers. By adding these fillers, the conductivity of the material was increased, enabling the SME to be triggered through Joule heating. The SME was activated through thermostable mechanisms that involve the presence of covalent bonds. When heated above the ionic *T*_*S*_, these mechanisms activated the SME in the materials, rendering ionic interactions ineffective. Consequently, the material could be deformed into a new shape, which remained stable until heated above this threshold temperature again. Upon cooling, these ionic interactions were reactivated and their temporary shape was preserved until the material was subjected to further heating. The shape memory elastic composite exhibited excellent SME, with both the shape recovery rate and shape fixation rate exceeding 90%.

Electro-responsive two-way shape memory elastomers are smart materials capable of bidirectional reversible actuation when stimulated by a current. However, there have been few studies on the effect of electro-responsive 2W-SME due to the limitations of these materials. Recently, a novel semi-crystalline thermoplastic polyamide elastomer (TPAE) with a 2W-SME was reported.^92^ MWCNTs provided 2W-SME in the TPAE (T12) material, exhibiting excellent conductivity (Figure 9a). The CNTs did not significantly influence the 2W-SME of the T12/CNT composites. Furthermore, they created a dual-layer actuator using SEBS and T12/CNT-5 composites to enhance the electro-responsiveness of the 2W-SME (Figure 9b). The 2W-SME of the composites were shown in Figure 9c. The Joule heating generated by the conductive CNTs network in the composite enabled the shape recovery temperature to be reached (Figure 9d), ranging from 36 to 90 °C. Figure 9e shows the electro-responsive two-way shape memory process of double-layer SEBS/T12/CNT-5. When the power was turned off and on, the angle of the dual-layer SEBS/T12/CNT-5 actuator ranged from 18 to 21°, with approximately 60% of the change occurred in the first 2 min after switching the power supply on or off.

**Figure 9:**
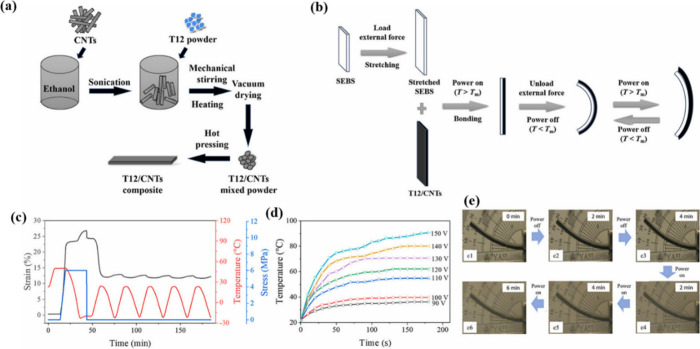
(a) Technical process for preparing T12/CNTs. (b) Bilayer actuator composed of T12/CNTs-5 and SEBS. (c) Reversible two-way shape memory curve of the T12/CNTs-5 composite. (d) Surface temperature changes of T12/CNTs-5 under varying voltages. (e) Electro-induced 2W-SME of bilayer SEBS/T12/CNTs-5 actuator at 110 V. (a–e) Adapted with permission from ref 92. Copyright 2024 Elsevier.

### 3.4 Magneto-Responsive Shape Memory Elastomers

Magneto-responsive shape memory elastomers are a special type of polymer materials with magnetic responsiveness and shape memory properties.^93^ These materials undergo shape transformation in response to an external magnetic field and restore their initial shape upon the removal of the magnetic field, showing high controllability and reversibility. The addition of magnetic nanofillers can induce magneto-responsive SME, thereby presenting a crucial concept for developing such materials.^136^ Magnetic nanoparticles (MNPs) convert electromagnetic energy into heat under alternating magnetic fields.^137^ Thus, the increased temperature of the MNPs can activate shape memory switches, enabling them to regain their shape.

Babaie et al.^94^ suggested modifying Fe_3_O_4_ MNPs with octadecyl isocyanate (OD) to enhance their affinity with a PU matrix via covalent urea linkages, and added it as a magnetic nanofiller into shape memory elastomers to fabricate magneto-responsive shape memory elastic composites (Figure 10a). The nanocomposites displayed a thermoresponsive SME, exhibiting high *R*_*r*_ and *R*_*f*_ values, which were attributed to the crystallizable PCL segments. As shown in Figure 10b and c, heat dissipation form the MNPs under an alternating magnetic field had the potential to elevate the temperature of the sample above its switching temperature (*T*_*S*_ = *T*_*m‑PCL*_). As a result, SME was realized by activating shape memory switches. Analysis of the results revealed that these samples were exposed to an alternating magnetic field, and the temperature of the nanocomposites could rise to approximately 42 °C, with shape recovery occurred within 90 s. It should be noted that the shape programming process was achieved by heating directly above its *T*_*S*_, and the shape recovery process was activated by an alternating magnetic field (Figure 10d). The shape fixation and recovery rates of the nanocomposites exceeded 95% at 50 °C.

**Figure 10:**
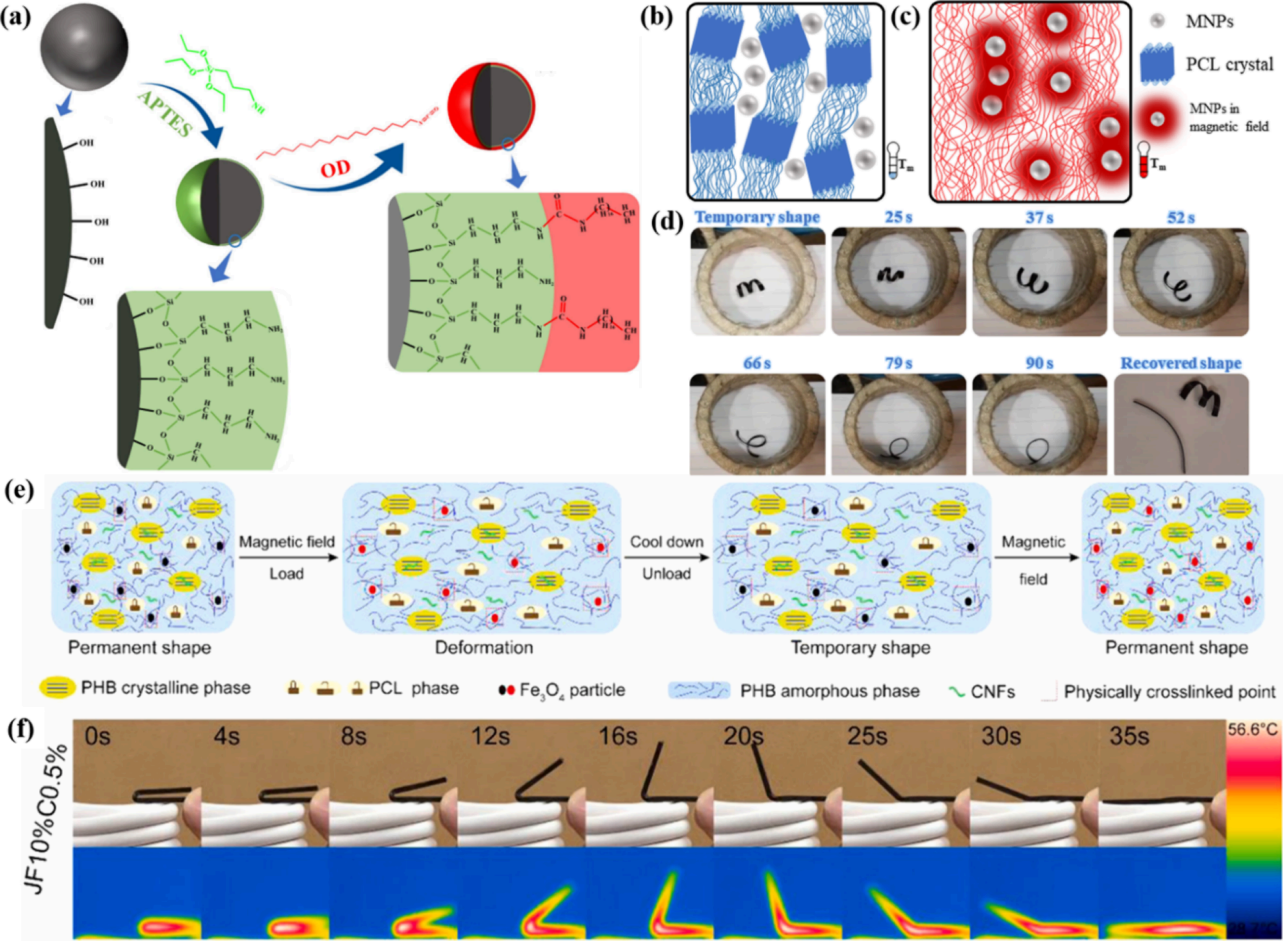
(a) Molecular structure for unmodified, OD and (3-aminopropyl) triethoxysilane-grafted, OD-grafted MNPs. (b, c) Melting of PCL crystals with the heat dissipation of MNPs in alternating magnetic field (b) (*T*_*low*_ <*T*_*m*_) and (c) (*T*_*high*_ > *T*_*m*_). (d) Magneto-responsive shape recovery process. (e) Magneto-responsive SME mechanism of diagram composites. (f) Magnetic-field-triggered SME. (a-d) Adapted with permission from ref 94. Copyright 2022 Wiley. (e, f) Adapted with permission from ref 95. Copyright 2021 Elsevier.

In addition, magneto-responsive shape memory composites (SMCs) with excellent strength and toughness can be achieved by incorporating two fillers. Yue et al.^95^ fabricated magneto-responsive SMCs by incorporating Fe_3_O_4_ and cellulose nanofibers (CNFs) into poly-hydroxybutyrate/poly­(ε-caprolactone) blend systems. Fe_3_O_4_ vibrated under a magnetic field to generate heat. The PCL phase melted when the temperature reached *T*_*m‑PCL*_. After shape memory programming was completed, the temporary shape of the sample was stabilized by the PCL phase, and the physical entanglement points between the PHB crystal phase and Fe_3_O_4_ in the composite memorized the permanent shape of the samples (Figure 10e). Figure 10f illustrates the magnetic field-triggered shape recovery process of the specimens (*R*_*f:*_ 100%).

In recent years, shape memory elastomers activated by photothermal conversion and magnetic fields have been extensively studied. Unlike multi-responsive shape memory elastomers, materials commonly accomplish the programming and recovery processes of shape memory through two or more external stimuli. This phenomenon was known as synergistic control. Chen et al.^96^ developed novel light magnetic-responsive shape memory elastomers based on biocompatible PCL/TPU/Fe_3_O_4_@PDA nanocomposites. The Fe_3_O_4_@PDA NPs generated heat (*T*_*s*_) under light exposure. The *R*_*f*_ and *R*_*r*_ values were 94.1% and 82.3%, respectively. Following light exposure without a magnetic field, the cantilever returned to its original flat position (Figure 11a, b).

**Figure 11:**
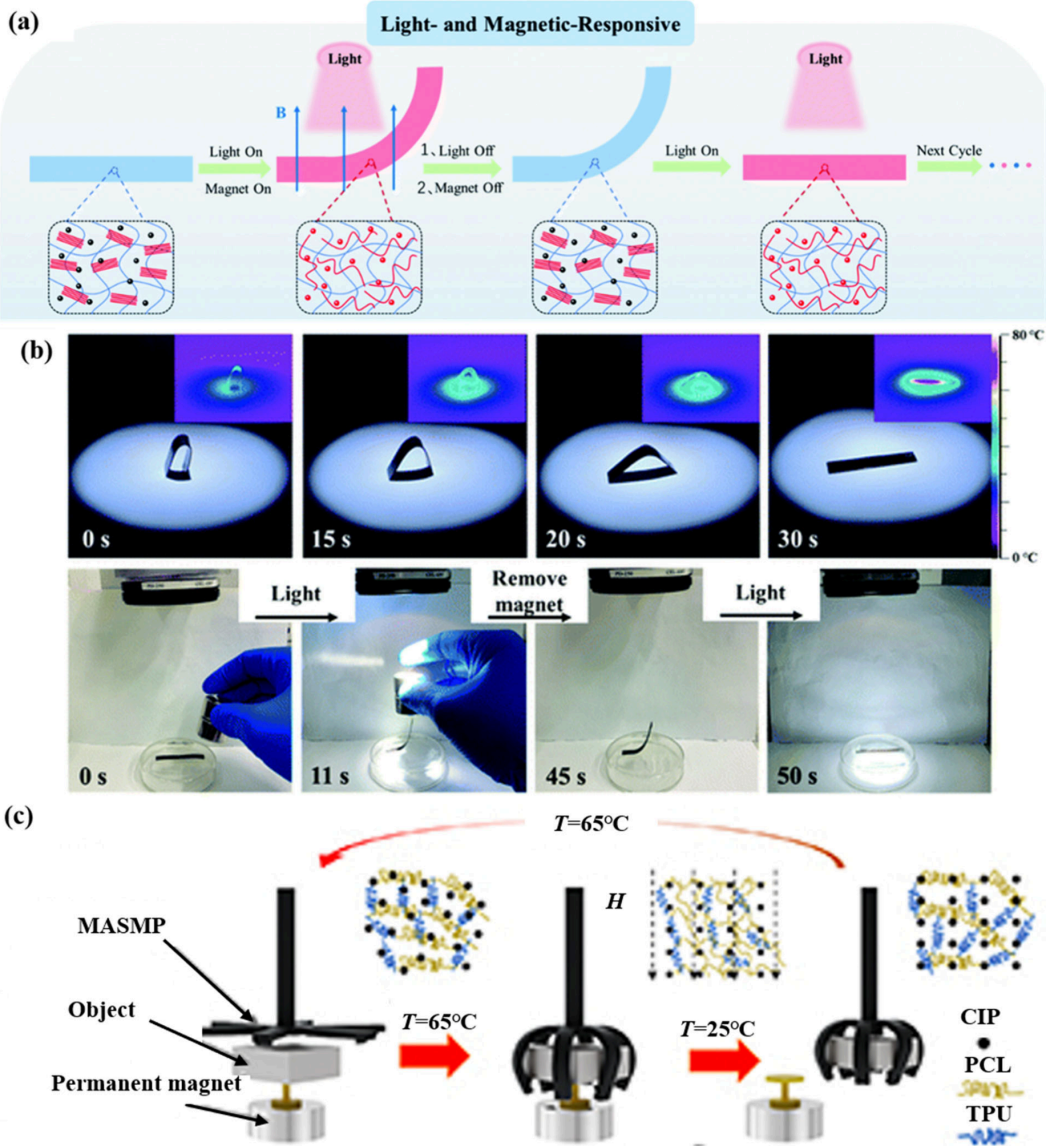
(a) Light- and magneto-responsive actuation. (b) IR thermal images of light-triggered shape recovery processes and the controlled reconfiguration response to both light and magnetism. (c) Grasping and releasing of magneto-driven flexible claw. (a, b) Adapted with permission from ref 96. Copyright 2021 Royal Society of Chemistry. (c) Adapted with permission from ref 97. Copyright 2023 IOP Publishing.

Thus, photothermal heating and magneto-responsive actuation achieved a reversible-shape transformation. In addition to using Fe_3_O_4_ particles as magnetic fillers, researchers have added iron carbonyl iron particles (CIPs) as magnetic fillers. Yao et al.^97^ prepared magneto-responsive SMCs by incorporating CIPs as magnetic fillers into a blend of polycaprolactone and TPU. In addition, they printed a flexible claw point using magneto-responsive shape memory elastomers as the raw material for 4D printing (Figure 11c). The magneto-responsive SMCs had excellent shape memory performance (*R*_*f*_, *R*_*r*_ > 95%).

### 3.5 Solvent-Responsive Shape Memory Elastomers

Thermoresponsive SMEs have been extensively studied, because of their straightforward triggering mechanisms. However, precise control over the switching temperature within a range suitable for biomedical applications remains challenging, restricting their widespread use in biomedicine.^138^ Alternatively, stimuli such as light, electricity, magnetism, and pH can pose risks and inconveniences in biomedical applications. Solvent, (i.e., water), being abundant in the body and inherently biocompatible, has emerged as a promising trigger for shape recovery effects, offering a potential solution to these challenges.

Liu et al.^139^ integrated the phase-transition-induced stiffening principle from shape memory metallic alloys into the molecular design of shape memory PUs. This design incorporated all-hard segments in the main chains combined with PEG with dangling side chains (Figure 12a). Shape recovery was achieved by a combination of heat and water. Figure 12b illustrates the domain morphologies of SPPU4 (PUs with the hard segment as the main chain and the hanging PEG chains as the soft segment) and PPU4 (PUs with PEG soft segments on the main chains) in both dry and hydrated states. The SPPU6 shape recovery process were presented in Figure 12c. The *R*_*r*_ of SPPU6 was 42.1% at 37 °C in a dry state but increased to 71.5% when immersed in water at the same temperature. SPPU4 exhibited an even better shape recovery performance, achieving an *R*_*r*_ of approximately 87.2%.

**Figure 12:**
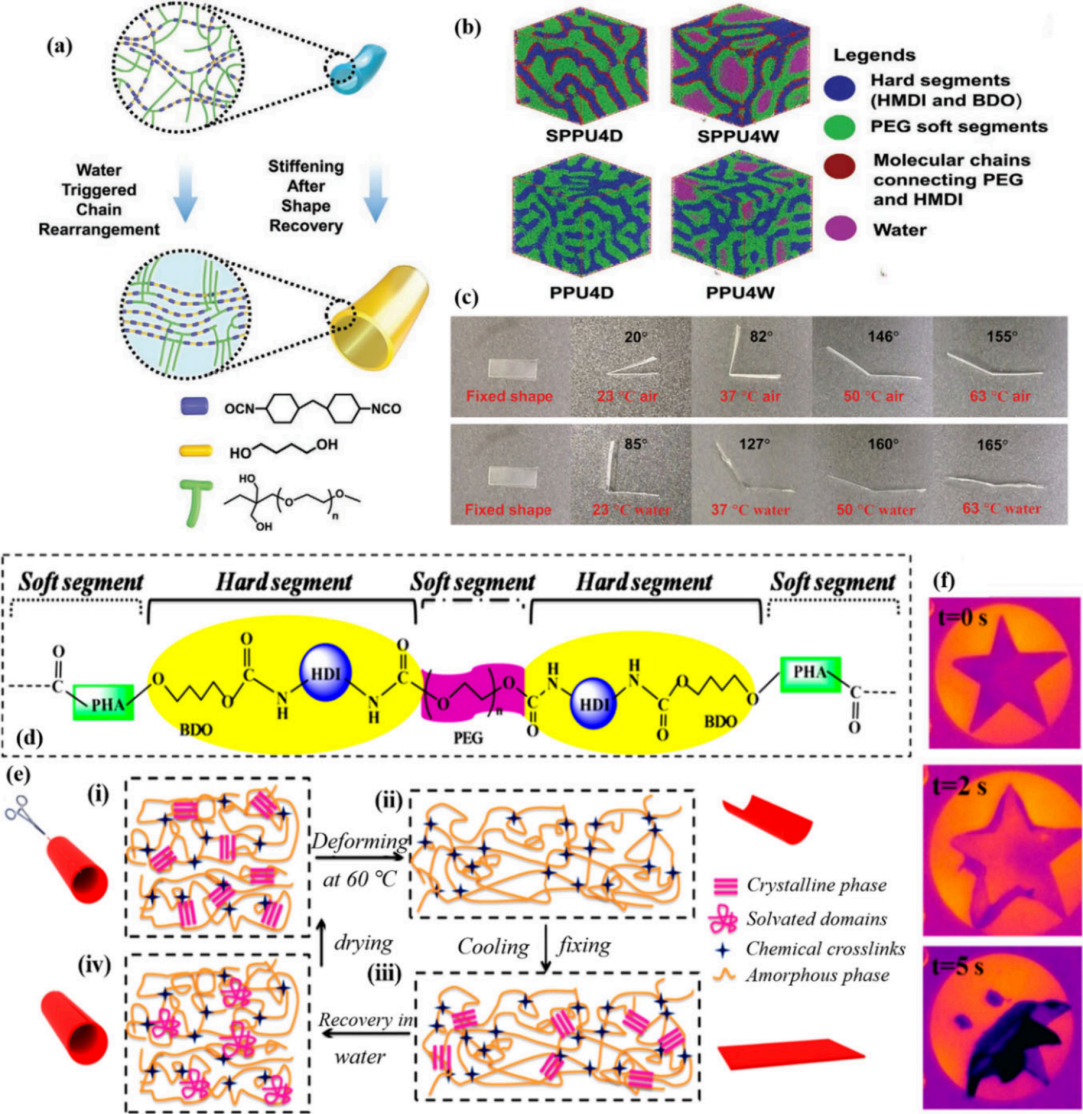
(a) Water-induced stiffening of shape memory elastomers during shape recovery. (b) Microphase morphology of shape memory elastomers under dry and hydrated conditions. (c) Shape recovery of SPPU6 in both air and water. (d) Structure of polyhydroxyalkanoate (PHA)-based polyurethane (PHP). (e) Mechanism for the shape memory transition. (f) State after 5 s soaking in water at room temperature. (a–c) Adapted with permission from ref 139. Copyright 2022 Wiley. (d–f) Reproduced from ref 98. Available under a CC-BY 4.0 license. Copyright 2019 Wang et al.

In addition, there are shape memory elastomers with water/thermal dual-responsive SME. Wang et al.^98^ produced a new type of bio-based material using polyhydroxyalkanoate (PHA), which had an excellent SME. Telechelic-hydroxylated PHA and PEG were utilized as soft segments of PHA-based polyurethane (PHP), providing thermoresponsive and water-responsive regions, respectively (Figure 12d). Figure 12e illustrates the mechanism of the water-responsive SME in the PHP film. Thus, the PHP exhibited excellent thermal and water responses with high *R*_*f*_ (>99%) and *R*_*r*_ (>90%) values. As shown in Figure 12f, a pentacle membrane made of PHP rapidly deformed in water at room temperature within 5 s. This indicated that PHP had water-heat dual-responsive shape memory properties.

Water-responsive shape-memory composites have also been discussed in recent reports. Majdoub et al.^99^ developed water-responsive shape-memory nanocomposites by adding graphitic carbon nitride nanosheets (g-C_3_N_4_) to poly­(vinyl alcohol) (PVA) films. Figure 13a shows the mechanism of the PVA/g-C_3_N_4_ water-responsive shape-restoration behavior. The water-responsive SME of PVA-filled g-C_3_N_4_ nanocomposites (PVA/g-C_3_N_4_) was significantly improved by the formation of robust hydrogen bonds between the uncondensed amine groups of g-C_3_N_4_ and the hydroxyl groups of PVA. These interactions lead to the formation of additional physical cross-links. As shown in Figure 13b, the PVA/g-C_3_N_4_ 1 nanocomposites quickly revert to their initial shapes upon immersion in water at room temperature (*R*_*r*_ >80%).

**Figure 13:**
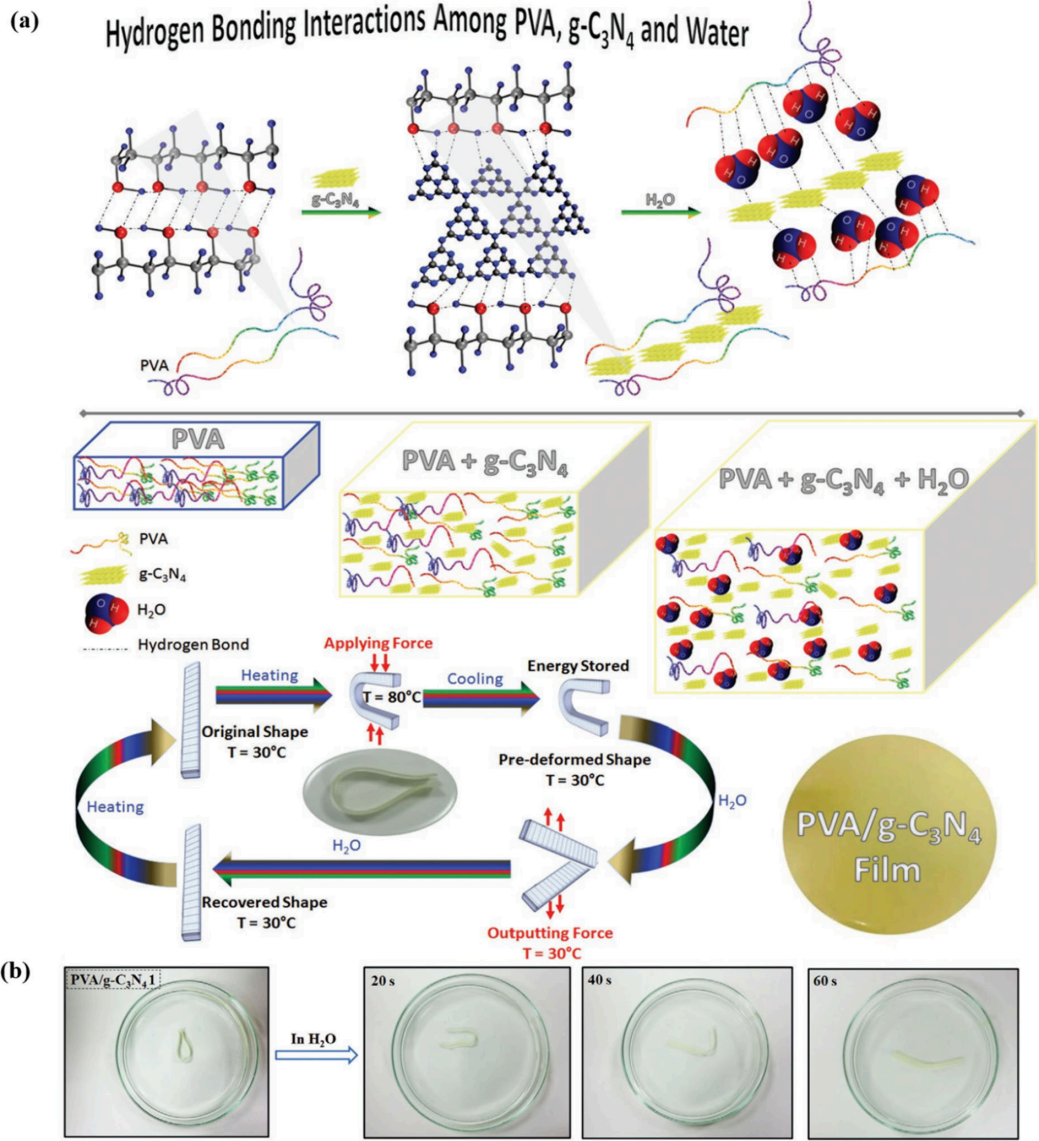
(a) Incorporating hydrogen bonding among PVA, g-C_3_N_4_, and water (above), and the water-induced shape memory mechanism (below). (b) Water-responsive SME of PVA/g-C_3_N_4_ 1 films in water. (a, b) Adapted with permission from ref 99. Copyright 2022 Wiley.

Researchers have prepared water-responsive shape memory elastomers using one way-triple SME and 2W-SME. Liang et al.^100^ fabricated water-responsive shape memory elastomers using polytetrahydrofuran (PTHF) and hydrophilic PEG oligomers. Interestingly, the 2W-SME was triggered by body temperature, whereas the one way-triple SME was triggered by water and body temperature. These innovative features have enabled polymers to play significant roles in biomedical applications. The partial PEG chain segment at a lower *T*_*m*_ and the PTHF chain segment provided reversibility to the 2W-SME (Figure 14a). The PEG chain segment in the higher *T*_*m*_ region was responsible for programming the anisotropy. Figure 14b and c provided examples of programmable 3D structures, such as flowers and towers, which could be reversibly actuated using papercutting technology, resulting in shape programming at 37 °C and shape reversibility at −15 °C. The hydrophilicity of the PEG segment conferred responsiveness to water. Therefore, by leveraging the temperature sensitivity of the PTHF segment and water responsiveness of the PEG segment, the polymer network exhibited a one way-triple SME (Figure 14d). A ribbon polymer sample with a composition of P60-G40 was shaped into temporary shape A at 75 °C, and upon cooling to 5 °C, shape A was stabilized through the crystallization of the PEG phase. By additional deformation and subsequent cooling to −20 °C to induce PTHF crystallization, temporary shape B could be obtained. The temporary and initial shape could be recovered by heating and water treatment.

**Figure 14:**
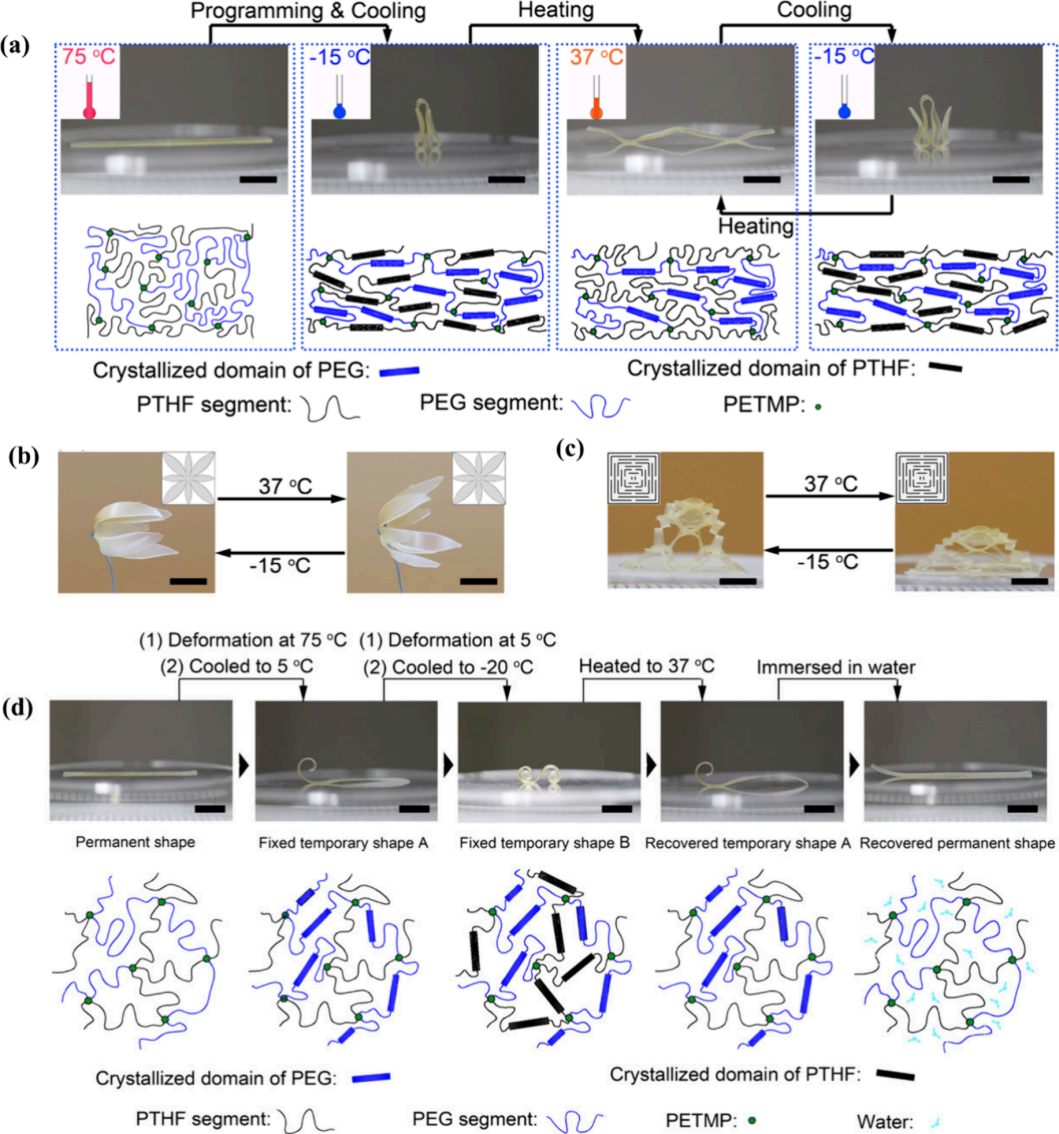
(a) Shape programming, reversible actuation, and the network structure. Reversible actuation of programmed (b) 3D flower and (c) 3D tower. (d) Body-temperature- and water-responsive triple SME and network structure. (a–d) Reproduced from ref 100. Copyright 2021 American Chemical Society.

### 3.6 Multi-Responsive Shape Memory Elastomers

Multi-responsive shape memory elastomers are a category of smart materials characterized by their ability to respond to multiple stimuli simultaneously and exhibit versatile responsiveness to two or more triggers. These stimuli include the heat, light, electric fields, magnetic fields, and humidity. Compared with traditional single-stimulus-responsive shape memory elastomers, multi-responsive shape memory elastomers have a wider response range and more diverse application scenarios. It should be noted that the multi-responsive shape memory elastomers mentioned here refer to shape memory elastomers with three responses. Note that the multi-responsive shape memory elastomers mentioned here refer to shape memory elastomers with three responses. Du et al.^140^ investigated shape memory composites composed of POEs, lauric acid (LA) and carbon black (CB). This composite material exhibited a triple-stimulus-responsive SME.

LA with crystalline small molecules (*T*_*m*_ ≈ 50°C) was used instead of a semi-crystalline polymer, and it cooperated with POE to act as a fixing phase. The specific dispersion of the CB nanoparticles within the POE phase led to the creation of robust conductive pathways and increased the recovery force. LA, in combination with a small crystalline molecule, served as a trigger for shape transformation. As conductive and reinforcing fillers, CB nanoparticles enabled the composites to change shape and recover rapidly. POE/LA/CB (50/50/10) exhibited the best SME in the fifth cycle, achieving 95% *R*_*f*_ and 96% *R*_*r*_. Its thermoresponsive SME was shown in Figure 15a, recovering to its initial shape within 10 s at 65 °C. The shape recovery process under 10 V applied by DC power was shown in Figure 15b, with shape recovery in 45 s when the voltage rose to 15 V and in 20 s when the voltage rose to 20 V. Figure 15c illustrated solvent-activated SME, in which the material recovers its initial shape (<30 min) in ethanol, obviating the requirement for heating.

**Figure 15:**
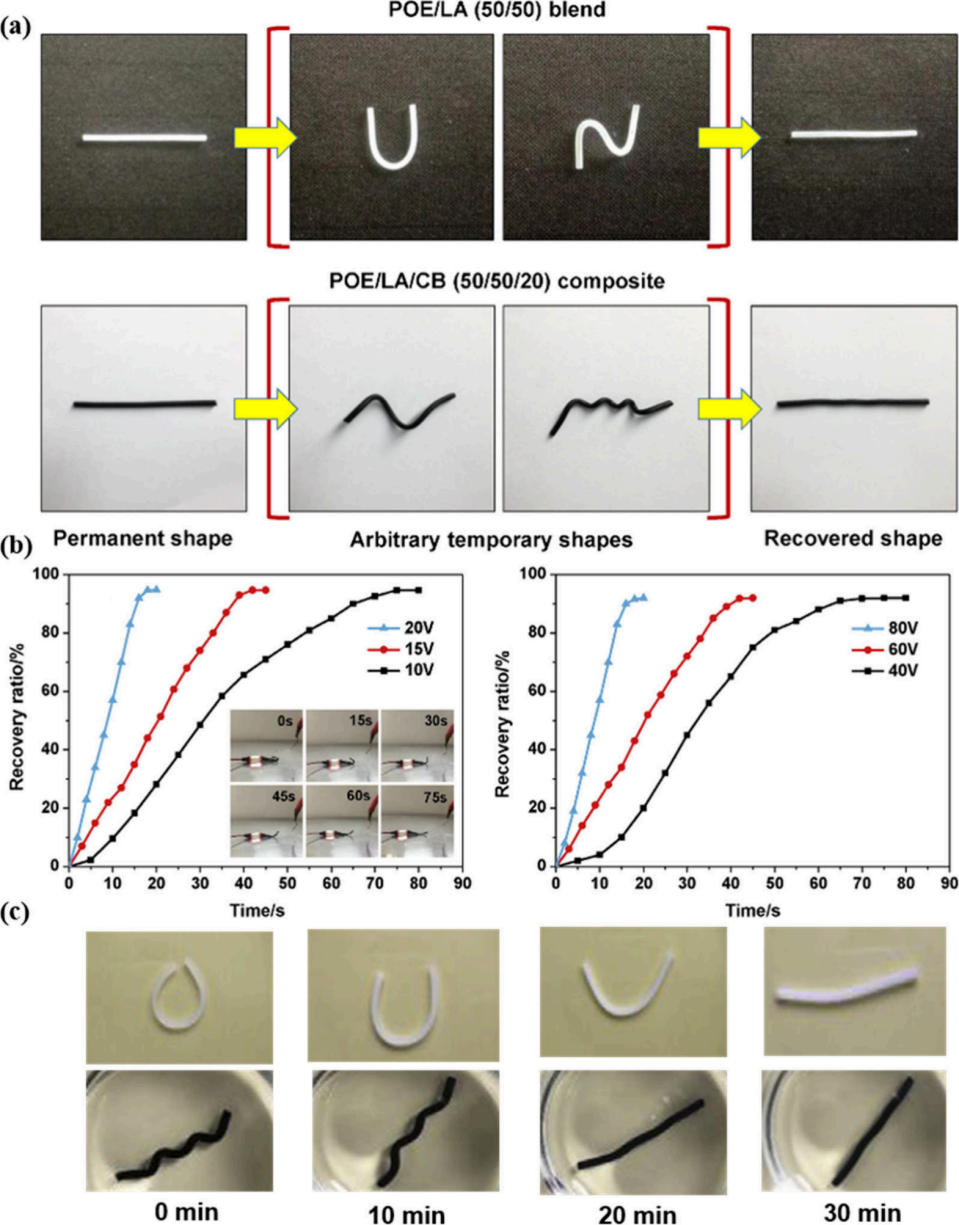
(a) SME of POE/LA (50/50) and POE/LA/CB (50/50/20). (b) *R_r_* over time for POE/LA/CB (50/50/20) (inset photos show electro-responsive POE/LA/CB (50/50/20) at 10 V) and POE/LA/CB (50/50/10). (c) POE/LA (50/50) (upper images) and POE/LA/CB (50/50/20) (lower images) in ethanol at room temperature. (a–c) Adapted with permission from ref 140. Copyright 2019 Elsevier.

Huang et al.^141^ designed thermoplastic vulcanizates (TPVs) with shape memory capabilities through dynamic vulcanization, enabling them to respond to multiple stimuli. These TPVs consisted of poly­(lactic acid) (PLA), ENR, and Fe_3_O_4_. PLA acted as the reversible phase, making the cross-linked ENR highly resilient. The dispersion of Fe_3_O_4_ in the two phases was uniform, which enhanced the rubber and interfacial compatibility.

Furthermore, the incorporation of Fe_3_O_4_ enabled the TPVs to exhibit distinctive shape memory properties that were responsive to alternating magnetic fields and NIR light at 808 nm. The sample P7E3F3 demonstrated the highest recovery ratios in two cycles, with 99.25% for *R*_*f*_ and 93.47% for *R*_*r*_, while the *R*_*r*,*induced*_ values of P7E3F3 were 97.72% in an alternating magnetic field and 96.05% in NIR. The multiple-SME was achieved by a *T*_g_ (70 °C) mechanism (Figure 16a). Figure 16b illustrates the shape recovery process at 70 °C. All TPVs exhibited rapid initial recovery within the first 5 s, followed by a gradual decrease in recovery speed. Fe_3_O_4_ could efficiently heat the matrix to the *T*_g_ of PLA, facilitating both the initiation and the completion of shape recovery (Figure 16c). Figure 16d shows the rapid shape recovery of P7E3F3 under NIR irradiation.

**Figure 16:**
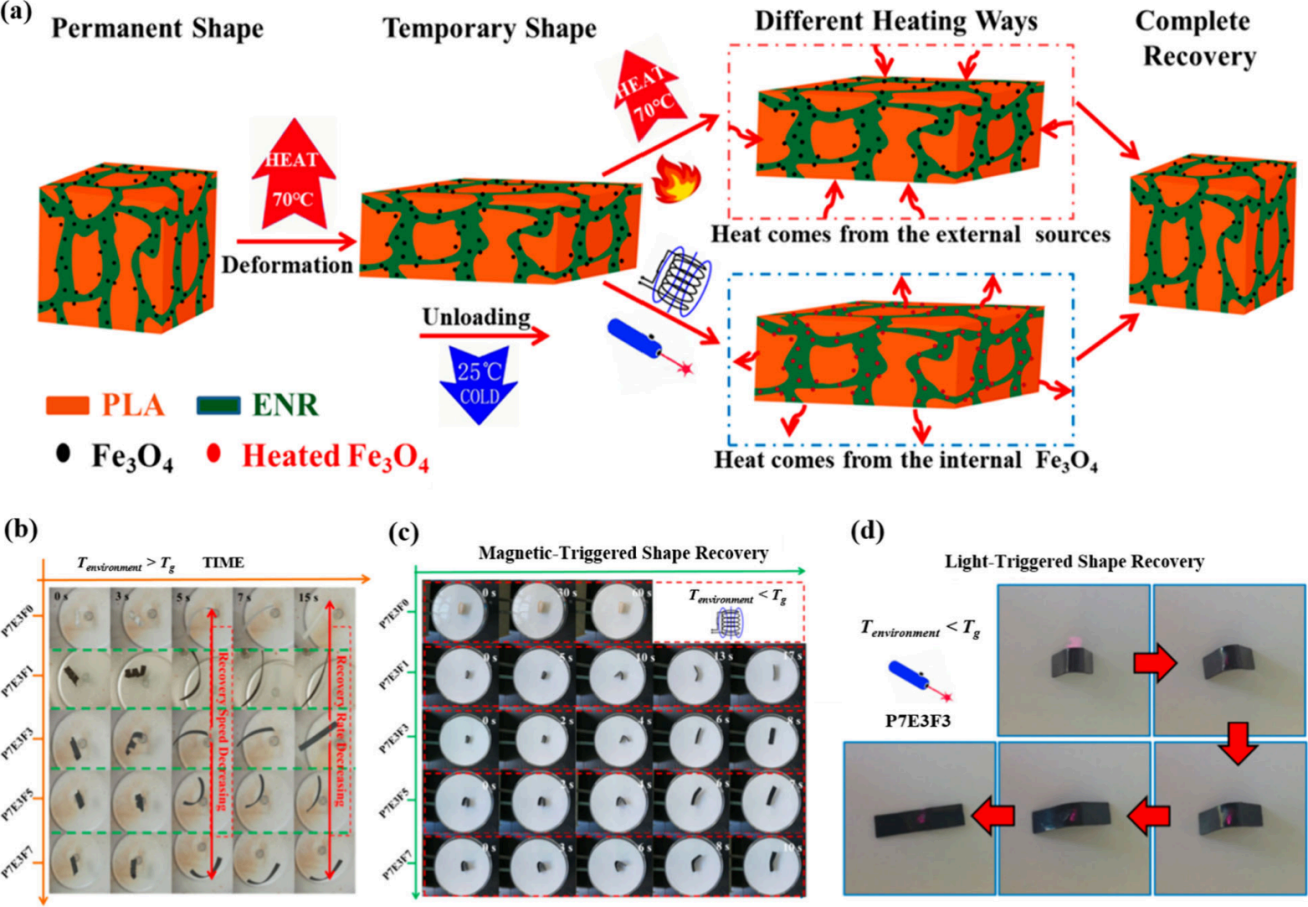
(a) Shape memory mechanism under different conditions. (b) Shape recovery of TPVs in water at 70 °C. (c) TPVs in an alternating magnetic field. (d) Shape recovery of P7E3F3 under NIR light. (a–d) Reproduced from ref 141. Copyright 2019 American Chemical Society.

Bayan et al.^142^ developed aliphatic hyperbranched PU nanocomposites that exhibited SME triggered by thermal energy (60 °C), microwaves (300 W), and sunlight (10^5^ lux). They utilized an aluminum-hydroxide-reduced graphene oxide nanohybrid as the key component. The hyperbranched PU (HPU) matrix, characterized by its unique structure of inherent hard and soft segments, along with rGO sheets incorporating nanohybrids, functions as a nanoheater, enabling effective shape memory performance. Multiple-SME was achieved using a *T*_*m*_ mechanism. The selected switching temperature was 60 ± 5 °C, closely the *T*_*m*_ of the polymer matrix. The shape was fixed as a spiral shape and was restored to a permanent shape after 7.3 min of sunlight exposure. In another example, the shape was fixed as a spiral and the permanent shape was restored using a thermal response stimulus shape. It took only 21 s to restore the permanent shape at 60 °C.

## 4 Applications of Shape Memory Elastomers

Shape memory elastomers exhibit elastic properties, excellent extensibility and recovery ability during deformation, which makes them very advantageous in situations that require frequent deformation and shape recovery.^181^ Compared to shape memory polymers, the prominent feature of elastomers is its excellent elasticity, draft ability, and flexibility. Therefore, shape memory elastomers have broad application prospects in the fields of biomedicine, soft actuators, artificial muscles, aerospace, shape-memory-assisted self-healing, and smart textiles.

### 4.1 Biomedical Field

Synthetic biodegradable elastomers, such as polyesters and polyurethanes, have transformed biomedical therapeutic approaches and devices.^143^ Owing to advancements in chemical synthesis and processing technologies, a range of biodegradable elastomers and their associated devices have been developed. These innovations have controllable properties and diverse functionalities, garnering substantial interest and demonstrating significant potential in biomedicine. Applications include self-tightening sutures, controlled drug release systems, and implants for minimally invasive surgery.^144^ Shape memory elastomers can be used as materials for 4D printing, which is a widely used technique. Most soft robotics, folding materials, biodegradable materials, medical devices, and other applications involve 4D printing. Therefore, shape memory elastomers play an important role in these fields. Paunović et al.^145^ reported the 4D printing of biodegradable shape memory elastomers with tailorable transition points covering physiological temperatures. They achieved the ideas by using poly­(d,l-lactide-*co*-trimethylene carbonate) methacrylates with varying monomer feed ratios (Figure 17a). The printed elastomers were biocompatible and degraded within 2.5 months under physiological conditions, confirming their potential for the manufacturing of smart medical devices. Aliphatic polyesters have been frequently utilized as scaffolds in tissue engineering owing to their excellent biodegradability and biocompatibility. Luo et al.^146^ used fused deposition modeling (FDM) 4D printing of cross-linkable linear shape memory copolyesters to fabricate scaffolds with fine structure (Figure 17b). Under UV irradiation, the 4D models exhibited outstanding SME, mechanical performance, and stability in water, facilitated by chemical bonding between each layer.

**Figure 17:**
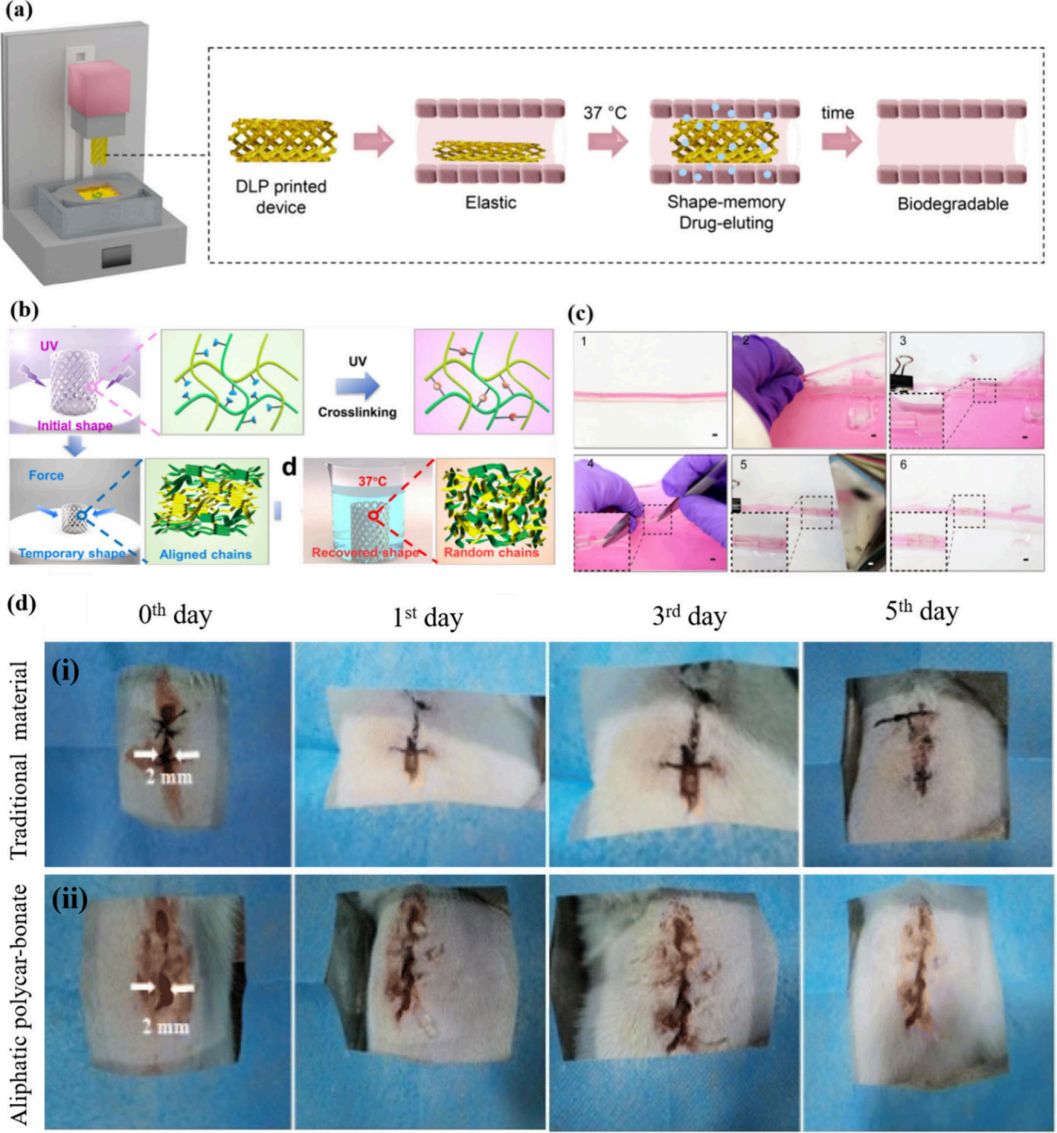
(a) 4D-printed biodegradable drug-delivery device with elasticity. (b) 4D-printed biological tissue engineering scaffolds. (c) Potential application of SM tubing. (d) Heat irradiation suture for wound closure: the wound healing process over time, illustrating the progression of healing after the wound edges are tightened at room temperature. (a) Reproduced from ref 145. Available under a CC-BY 4.0 license. Copyright 2023 Paunović et al. (b) Adapted with permission from ref 146. Copyright 2023 ACS Publications. (c) Reproduced from ref 147. Copyright 2018 American Chemical Society. (d) Adapted with permission from ref 149. Copyright 2023 Royal Society of Chemistry.

Moreover, the 3D printing of flexible materials with smart capabilities, such as shape memory, is crucial for advancing the 4D printing technology across various applications. Kuang et al.^147^ developed an ink comprising urethane diacrylate and a linear semi-crystalline polymer. The resulting 3D-printed shape memory elastomers showed promise for biomedical applications, such as vascular repair devices (Figure 17c). This study opens new avenues for the advancement of 4D printing.

Shape memory elastomers can be manufactured into surgical sutures to promote wound healing. Chen et al.^148^ reported a photothermal-responsive elastomer composite material that used its self-healing ability and shape memory as sutures to promote wound healing. The self-tightened suture rapidly returned to a predetermined shape at physiological temperatures, effectively sealing the injured wound surface. Sun et al.^149^ synthesized a biodegradable aliphatic polycarbonate intelligent elastomer. The elastomer exhibited outstanding biocompatibility, establishing them as suitable biomedical materials. Subsequently, in vivo experiments were conducted to validate the self-healing knotting capabilities of the polymers, which enabled them to rapidly close the wound surface by adopting a programmed shape at physiological temperatures (Figure 17d). Liu et al.^150^ developed a series of bioderived PUs that exhibited dual, triple, and quadruple SME. In addition, the multiple dynamic interactions between polymer chains provided PUs with good self-healing properties and better biodegradability than nylon sutures. Biological sutures made from PUs achieved clinical healing after 10 days.

### 4.2 Soft Actuator and Robots

Soft actuators are a type of driving device based on soft materials and are typically used in robots, bionic systems, and other mechanical equipment. Compared with traditional rigid actuators (such as motors or hydraulic systems), soft actuators have higher flexibility and adaptability. Du et al.^151^ reported a series of soft actuators that can be stimulated by a finger touching the palm, gentle blowing, or dim NIR light. These actuators were fabricated via the assembly of a reversible bending bilayer structure consisting of a film of a metallo-supramolecular dynamic network and kraft paper (Figure 18 a–c). The reversible bending behavior of the bilayer-structured material was enabled by the reversible SME of the synthetic polymeric material film, which converted molecular chain movement into macroscopic autonomous motion. This represented a long-sought-after and otherwise unattainable strategy. Ren et al.^152^ reported water-responsive actuators based on bilayer composite films fabricated from commercial PVA and SBS via a two-step solution-casting method. The resultant bilayer film exhibited a water-responsive 2W-SME and excellent cycling performance over five cycles. Flower-like and claw-like actuators based on a bilayer composite film were prepared, and their actuation performance was demonstrated (Figure 18d, e), suggesting their potential for applications in the fields of biomedicine and sensors.

**Figure 18:**
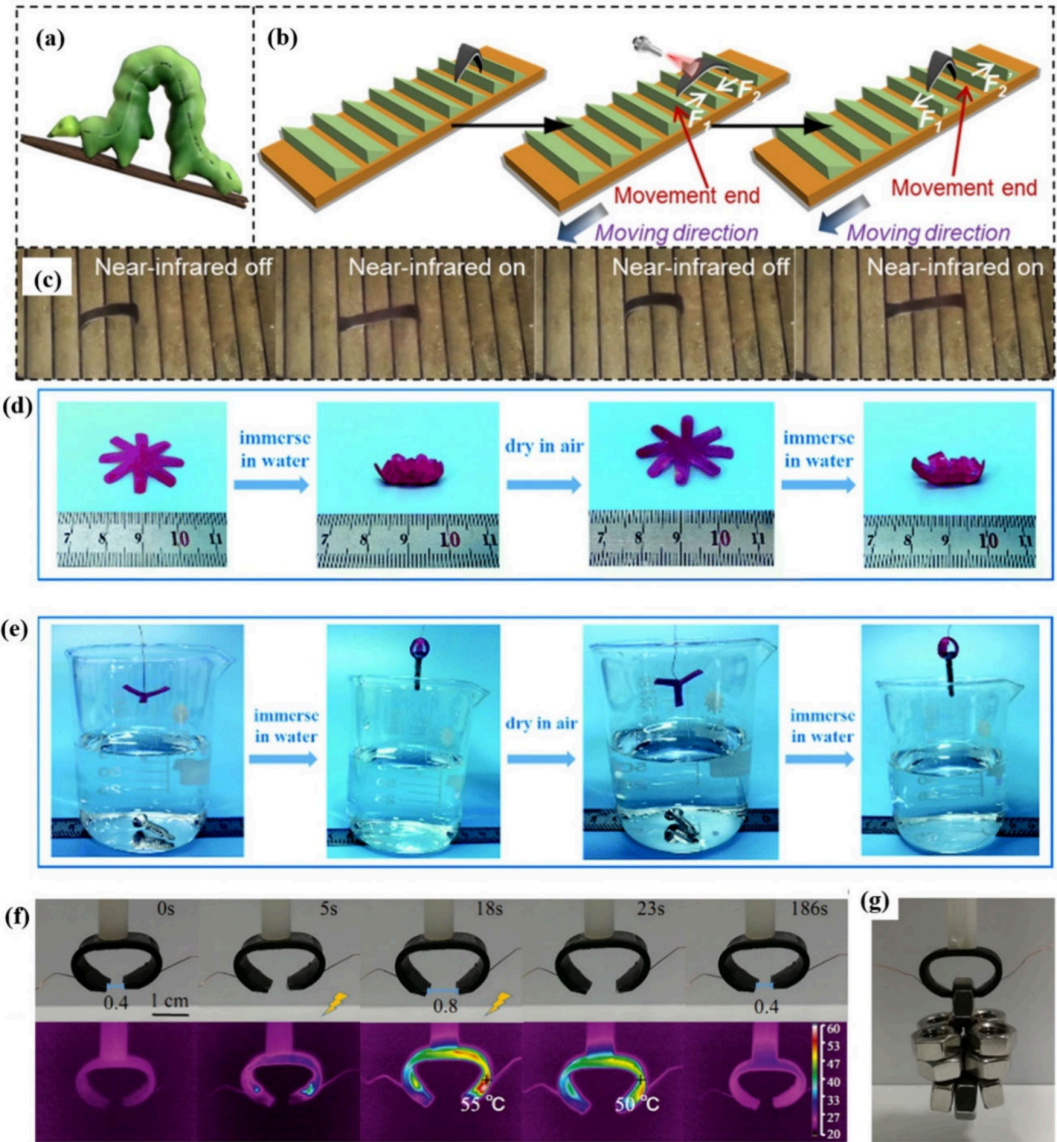
(a) Picture of a caterpillar. (b) Stress analysis for an NIR-light-driven soft-robot caterpillar. (c) NIR-light-driven soft-robot caterpillar crawling along a dentate substrate with or without NIR light. (d) Flower-like and (e) Claw-like actuators. (f) Digital and corresponding IR pictures of mechanical gripper. (g) Gripper for grabbing nuts. (a–c) Adapted with permission from ref 151. Copyright 2020 Elsevier. (d, e) Adapted with permission from ref 152. Copyright 2020 Royal Society of Chemistry. (f, g) Reproduced from ref 154. Copyright 2019 American Chemical Society.

Moreover, soft robots are constructed by using flexible and deformable materials designed to imitate the natural movement and adaptability of organisms. Jin et al.^153^ devised a strategy that uses a programmable crystalline shape memory polymer with thermo- and photo-reversible bonds to create a single-component robot. The global 3D-shaped structural support was fabricated using a plasticity-based origami technique enabled by thermo-reversible bonds. More critically, precisely controlled localized actuation could be programmed into 3D origami by using photo-reversible bonds. Overall, polymer thin films could be programmed into various soft robots, including 3D cranes and elephants. Xu et al.^154^ introduced a segregated structure of CNT in POE to fabricate multi-responsive reversible shape memory elastomers. The prepared materials could be driven at low voltage (≤36 V) with good driving performance. Moreover, the authors fabricated an electric gripper and a light-active crawling robot based on the prepared composites and demonstrated the potential applications of the POE/S-CNT composites in multiresponse-driven robots (Figure 18f, g).

### 4.3 Artificial Muscles

Artificial muscles are materials or devices that imitate the functions and movements of biological muscles. They are commonly used in robots, medical devices, and other applications that require flexible movement. Wang et al.^155^ designed and prepared a series of light-actuated 2W-SME composites by incorporating very small amounts of polydopamine (PDA) nanospheres into semi-crystalline polymer networks based on biodegradable poly­(ε-caprolactone) copolymers. The PDA nanospheres were well dispersed in chloroform, mixed well with the polymer network, and exhibited good photothermal effects owing to their strong absorption of light. This composite was capable of lifting and lowering weights, acting as an artificial muscle, and its actuated stress was significantly higher than the maximum stress yielded by most mammalian skeletal muscles.

### 4.4 Aerospace

The utilization of shape memory elastomers in aerospace applications presents a compelling and potentially advantageous opportunity, albeit alongside several significant challenges that must be addressed in the scope of their development and control, specifically related to the harsh and extreme conditions that are prevalent in outer space, such as extremely high or low temperatures, high vacuum, and exposure to intense UV radiation.^156^ These factors significantly reduce the effectiveness of shape memory applications without appropriate protective strategies.^157^ Therefore, the careful selection and development of shape memory elastomers for such applications is crucial. In particular, light- and electro-responsive shape memory elastomers provide a promising approach for remotely controlled actuators suitable for various aerospace applications.^158^ Arun et al.^159^ developed an innovative design of electro-responsive PU incorporating 2–40 wt % CB, tailored specifically for aerospace applications. This material integrated both electromechanical and thermomechanical actuation to exhibit distinct multistimulus responsiveness, making it a superior choice for aerospace applications. A novel stable SEBS/paraffin composite phase change material (C-PCM) with self-healing properties, elasticity, and deformability was reported.^160^ Paraffin (RT52 and OP10E) served as the phase change material (PCM), with SEBS chosen as the carrier material for the physical cross-linking of paraffin. The latent heat was as high as 188.1 J/g, thermogravimetric and thermal cycling tests showed that it had excellent thermal stability; mechanical property tests revealed that it had good elastic properties, and the elongation after self-healing was as high as 1160%. A possible use would be as a flexible wearable device for astronauts.

### 4.5 Shape Memory-Assisted Self-Healing

Smart materials have received substantial attention, because of their unique features. Self-healing polymers are one category of smart materials.^161^ These polymers have the unique ability to heal spontaneously and autonomously after experiencing surface and internal damage.^162–167^ However, despite advancements in the study and development of self-healing polymers, significant issues remain such as complex preparation processes, diminished mechanical performance, and limited self-healing efficiency, which pose obstacles to their practical use and application.

Therefore, a simplified and cost-effective manufacturing process must be developed and implemented to produce elastomers with excellent mechanical strength and self-healing abilities. Researchers are eager to develop new materials that effectively combine the SME and self-healing abilities. Xie et al.^168^ synthesized supramolecular rubber elastomers using metal coordination between carboxyl-terminated polybutadiene and a polystyrene-vinylpyridine copolymer. Metal coordination interactions in shape memory-assisted self-healing was demonstrated (Figure 19a). Innovative shape memory-assisted self-healing was a promising solution for the swift and effective repair of severe cracks. Efficient dual-response shape memory-assisted self-healing elastic composites was reported.^169^ CNT/PCL/TPU composites used TPU as the polymeric matrix, PCL as a healing agent, and CNTs as a reinforcing and conducting network. Experiments confirmed that the composite material exhibited excellent shape memory, which helped to reduce the number of crack openings. Subsequently, the healing agent melt and filled the cracks (Figure 19b).

**Figure 19:**
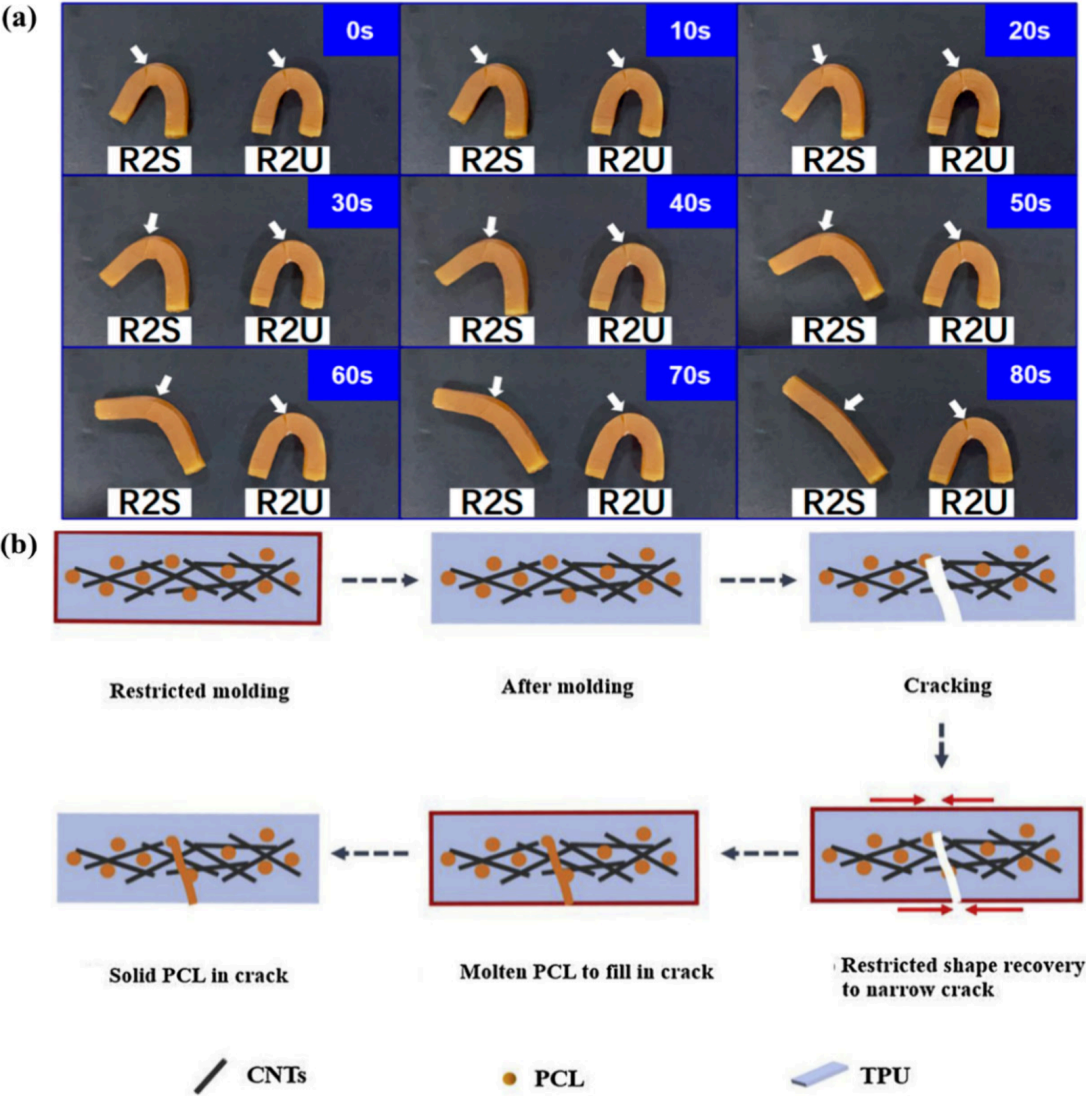
(a) Shape memory-assisted self-healing procedure. (b) Shape memory-assisted self-healing process. (a) Reproduced from ref 168. Available under a CC-BY 4.0 license. Copyright 2022 Xie et al. (b) Adapted with permission from ref 169. Copyright 2019 Elsevier.

### 4.6 Smart Textiles

Intelligent textiles are innovative products that combine traditional textile materials with advanced technologies. They perform the functions of perception, response, and interaction. Cheung et al.^170^ investigated the printing of thermoplastic polyurethane/elastomers (TPU/TPE) with varying Shore hardness (70 A, 82 A, 40 D) to improve the bond between thermoresponsive shape memory elastomers and nylon substrates. They found that Filaflex 70 A (TPU) exhibited stronger peel resistance with both a nylon substrate and shape memory elastomers. In addition, considering the comfort of the smart textile wearer, the textile material should have certain functional characteristics, such as permeability to water and air. Shape memory PU (SMPU) is an innovative functional material that continually enhances the aesthetic appeal and comfort attributes of derived products. SMPU could be the next candidate for smart textile materials in the next decade. Waterproof and breathable coated fabrics prepared using SMPU has been reported.^171^ Experimental data had consistently shown that in humidity-sensitive SMPUs, the fabric demonstrates reduced permeation, ensures adequate retention of body heat under low humidity conditions, and provides resistance to water droplets when required.

Given the increasing adoption of Internet of Things devices, there is a growing demand for smart materials and structures. Shape memory elastomers offer substantial opportunities for innovative applications in robotics, remotely operated systems, and packaging.

## 5 Summary and Outlook

In recent years, the rapid development of shape memory elastomers has created opportunities to improve their performance, according to the requirements of practical applications. This review covers recent research on thermo-, light-, electro-, magneto-, and solvent-responsive shape memory elastomers; the types of shape memory effects; different response mechanisms; and application prospects. Research on thermoresponsive shape memory elastomers is the most important. Light-, electro-, and magneto-responsive shape memory elastomers can be obtained by compositing light-thermal fillers (e.g., graphene) or introducing light-sensitive groups (such as azobenzene), conductive fillers (e.g., CNTs), or magnetic nanoparticles (e.g., Fe_3_O_4_). Consequently, most electro-responsive and magneto-responsive shape memory elastomers exhibit thermal responses. However, the solvent-responsive shape memory elastomers rely primarily on ingenious molecular-structure designs to achieve shape memory behavior.

Considering recent research, shape memory elastomers have tremendous potential for development. On the one hand, new shape memory elastomer structural designs are constantly being explored; on the other hand, the current properties of shape memory elastomers still need to be improved, which hinders their further applications. However, the current understanding of the mechanisms and design principles of shape memory elastomers is relatively complete, especially for simple one-way shape memory elastomers. The more complex the memory effect of shape memory elastomers, the greater the need for further understanding. For example, few studies have been focused on multi-responsive shape memory elastomers. 2W-SME is common in thermoresponsive shape memory elastomers but less common in other stimulus-responsive shape memory elastomers.

In future research, challenges will persist in many areas. First, special attention should be paid to the synthesis and enhancement of two way shape memory elastomers. Although the use of 1W-SME in some applications is mature enough, they are not suitable for applications in fields such as soft robotics and flexible electronics. Therefore, 2W-SME is a future research trend. The goal is to develop two way shape memory elastomers with a recovery stress potential comparable to that of shape memory alloys. Second, achieving precise control of shape recovery behavior is another important topic that has significant implications for the use of shape memory elastomers. This would lead to improvements in material functionality, reliability, and economy while concurrently propelling related fields towards expansion and innovation. Precise control of shape memory recovery behavior can achieve precise shape adjustment, so that materials can adapt to different application needs. For example, in medical devices, the shape of the bracket or implant can be customized according to the specific situation of the patient, thereby improving the treatment effects and patient comfort. In addition, most temporary shapes programmed in shape memory recovery process of shape memory elastomers are relatively simple spirals or folded shapes. In future research, 3D printing methods can be applied to achieve more complex temporary shapes. Using this method, the application field of shape memory elastomers can be significantly broadened. Finally, there is often a contradiction between the elasticity and shape recovery ability of shape memory elastomers. For example, enhancing the performance of shape recovery (such as through a high cross-linking degree or an enhanced phase transition effect) may lead to reduced elasticity, and increasing elasticity often affects the triggering and recovery speed of the shape memory effect. How to establish a balance between the two aspects is still a challenge. In practical applications, shape memory elastomers need to withstand complex external loads. How to improve its mechanical properties under various stress states such as tension, compression, and shear to ensure that premature fatigue and permanent deformation do not occur during long-term use is also a problem. Although shape memory elastomers face many potential challenges, their future development is still full of hope and opportunities.

## #

M. Q. Zhang: Deceased on December 20, 2024.
